# Repeated trans-arterial treatments of LDL-DHA nanoparticles induce multiple pathways of tumor cell death in hepatocellular carcinoma bearing rats

**DOI:** 10.3389/fonc.2022.1052221

**Published:** 2022-11-24

**Authors:** Yuzhu Wang, Junjie Li, Goncalo Dias do Vale, Jaideep Chaudhary, Arnida Anwar, Jeffrey G. McDonald, Tao Qin, Hongwei Zhang, Ian R. Corbin

**Affiliations:** ^1^ Department of Hepatobiliary and Pancreatic Surgery, Henan Provincial People’s Hospital, Zhengzhou University People’s Hospital, Henan University People’s Hospital, Zhengzhou, Henan, China; ^2^ Advanced Imaging Research Center, University of Texas Southwestern Medical Center at Dallas, Dallas, TX, United States; ^3^ Center for Human Nutrition and Department of Molecular Genetics, University of Texas Southwestern Medical Center at Dallas, Dallas, TX, United States; ^4^ Internal Medicine Division of Liver and Digestive Diseases, University of Texas Southwestern Medical Center at Dallas, Dallas, TX, United States; ^5^ Radiology, University of Texas Southwestern Medical Center at Dallas, Dallas, TX, United States

**Keywords:** hepatocellular carcinoma, trans-arterial infusion port, nanoparticle, low-density lipoprotein, docosahexaenoic acid

## Abstract

**Introduction:**

Repeated hepatic arterial delivery of therapeutic agents to the liver by percutaneously implanted port-catheter systems has been widely used to treat unresectable liver cancer. This approach is applied to assess the therapeutic efficacy of repeated low-density lipoprotein-docosahexaenoic acid (LDL-DHA) nanoparticle treatments in a rat model of hepatocellular carcinoma.

**Methods:**

N1S1 hepatoma bearing rats underwent placement of a percutaneously implanted hepatic artery port-catheter system and were allocated to untreated, control LDL-triolein (LDL-TO) or LDL-DHA nanoparticle infusions groups. Treatments were performed every three days over a nine day study period. MRI was performed at baseline and throughout the study. At the end of the study tissue samples were collected for analyses.

**Results and Discussion:**

Implantation of the port catheters was successful in all rats. MRI showed that repeated infusions of LDL-DHA nanoparticles significantly impaired the growth of the rat hepatomas eventually leading to tumor regression. The tumors in the LDL-TO treated group showed delayed growth, while the untreated tumors grew steadily throughout the study. Histopathology and MRI support these findings demonstrating extensive tumor necrosis in LDL-DHA treated groups while the control groups displayed minor necrosis. Molecular and biochemical analyses also revealed that LDL-DHA treated tumors had increased levels of nuclear factor-kappa B and lipid peroxidation and depletion of glutathione peroxidase 4 relative to the control groups. Evidence of both ferroptosis and apoptosis tumor cell death was observed following LDL-DHA treatments. In conclusion repeated transarterial infusions of LDL-DHA nanoparticles provides sustained repression of tumor growth in a rat hepatoma model.

## Introduction

Only an estimated 20% of hepatocellular carcinoma (HCC) patients are diagnosed at early stage, and thus amenable to curative surgical treatments (1). For majority of the patients with more advanced tumors limited within the liver (intermediate/advanced-stage HCC), transarterial therapies are widely used as a palliative treatment (2). This technique takes advantage of the dual blood supply to the liver (80% *via* portal vein and 20% hepatic artery) and the preferential hepatic arterialization of liver tumors (3). Administration of antineoplastic agents through the hepatic artery would enable higher drug concentrations within the tumor while minimizing systemic exposure (4). Current transarterial therapies, which include transarterial chemoembolization (TACE) and hepatic arterial infusion chemotherapy (HAIC). The former involves formulating a cytotoxic anticancer drug with an embolic agent to inflict cytotoxic insult as well as blocking off the blood supply to the tumor (5). HAIC as the name implies infuses chemotherapy through the hepatic artery to deliver high concentrations of drug into the feeding arteries of the HCC. HAIC is more commonly performed in Asian counties, especially Japan, where it has been shown to be an effective treatment for advanced HCC (6). While both methods take advantage of the first-pass effect in the liver, current evidence suggests that single cycle of TACE/HAIC may not be sufficient for effective treatment of HCC, thus repeated transarterial treatments are generally performed to control tumor spread (7–11). Multiple cycles of TACE/HAIC, however, exposes the surrounding healthy liver to repeated bouts of cytotoxic chemotherapy or potential ischemic hepatic injury which often leads to progressive liver failure (12–14). In addition, TACE causes permanent arterial occlusion that restricts transarterial access in future TACE sessions. This approach induces tumor hypoxia, and inadvertently stimulates the development of tumor-feeding collateral vessels, contributing to tumor refractoriness and tumor regrowth (15, 16). These findings highlight the urgent need for novel therapies against HCC.

Preclinical evaluations of rodent models plays a central role anticancer drug discovery. While laboratory rodent models offers practicality, ease and low cost, transarterial drug delivery in these models is challenging. Several labs can achieve one time access of the hepatic artery through abdominal laparotomy (17–19) or catheterization of peripheral arteries (carotid or femoral) followed by fluoroscopy-guided catheter advancement to the hepatic vasculature (20). Repeated administration to the hepatic artery presents an even greater challenge for preclinical investigators. In recent years some progress has been made in this area through the application of percutaneous implantable port-catheter systems in small animal studies (21–23). The port-catheter system allows for facile and safe repeated arterial infusions without the need of repeated invasive surgeries for vascular access.

To this end in the present study we aim to evaluate the therapeutic efficacy of repeated administrations of low density lipoprotein-docosahexaenoic acid (LDL-DHA) nanoparticles in a rat orthotopic model of HCC. Dietary intake of long chain omega 3 fatty acids like DHA has been shown to reduce the risk of HCC development in individuals with known hepatitis infection (24). DHA has been shown to induce multiple cell death pathways in tumor cells (25–28). Conversely, DHA has also been shown to provide anti-inflammatory hepatoprotective benefits in the setting of liver disease (29, 30). Furthermore, studies from our own group has shown that nanoparticle formulations of DHA can elicit marked anticancer effects in cell culture (31, 32), direct tumor injection (33) and *via* hepatic artery injection (HAI) (34). Collectively, these previous finding provide strong rationale and support for the present preclinical investigation.

## Materials and methods

### Preparation of LDL nanoparticles

Apheresis plasma of patients with familial hypercholesterolemia was collected, LDL was isolated using sequential density gradient ultracentrifugation (35). Triolein (TO) and unesterified DHA (Nu-chek Prep, Inc) were incorporated into LDL by the reconstitution method as described previously (31).

### Cell viability test

The N1S1 rat hepatoma cell line (ATCC, CRL-1603, Manassas, VA, USA) was cultured in Dulbecco’s Modified Eagle’s Medium (Sigma, D6429) supplemented with 10% fetal bovine serum and 1% penicillin-streptomycin. Cells were incubated at 37°C in a humidified environment containing 5% CO_2_. For viability test, cells were seeded in 96-well plates with 30000 cells/well and grown to 80-90% confluency. Prior to treatment all cells were cultured in serum free media overnight (~18 hours). After respective treatments with LDL nanoparticles, cell viability was measured at 72 hours with water soluble tetrazolium salt, WST-8 (CCK-8, Dojindo Molecular Technologies). Briefly, cells were incubated with WST solution for 2 hours at 37°C. A ThermoMax M5 microplate reader was used to measure the absorbance at 450 nm. The relative cell viability was expressed as a percentage of the control.

### Chemical and cell death inhibitor studies

To assess the pathway of LDL-DHA mediated cell death cell viability assays were also performed in the presence of selected cell death inhibitors (iron chelator, defiprone (DFP); caspase inhibitor, z-VAD-fmk; ferrroptosis inhibitor, liproxstatin-1. All drugs were purchased from Selleck Chemicals. For this assay all cells were pretreated for 3 hours with the inhibitors prior to the addition of LDL-DHA. Chemicals or cell death inhibitors were used at the following concentrations: DFP, 20-40µM; Z-VAD-FMK, 50-100µM; liproxstatin-1, 50-200nM.

### Western blot

Samples were lysed in 1x cell lysis buffer (9803, Cell Signaling) and protein concentration was determined using Pierce™ BCA Protein Assay Kit (Thermo Fisher). Equal amounts of protein were loaded for each sample on a polyacrylamide gel and separated using electrophoresis. Thereafter, proteins were transferred to PVDF Transfer Membrane (Immobilon) and incubated overnight with primary antibodies against Gpx-4 (1:500 dilution, sc-50497), Cleaved Caspase-3(1:1000 dilutions, Abcam ab2302) and β-actin (1: 1000 dilution, Cell Signaling sc-47778). Horseradish peroxidase-conjugated (HRP-conjugated) secondary antibodies were used and western blot signals were detected with ECL (Bio-Rad Laboratories).

### Methods of lipid peroxidation measurement for N1S1 cells

N1S1 cells (350,000) in 2 ml media without serum were plated on 6 well plate and incubated overnight. The next day, cells were stained with 1 uM Bodipy C11 581/591 dye (Invitrogen) for 30 minutes at 37°C. After staining, the cells were treated with different concentration of LDL-DHA for 24 hours. Non adherent cells in media were collected and attached cells were trypsinized. All cells were pooled together and then washed with PBS. Bodipy fluorescence of the cells were measured with flow cytometry where green fluorescence indicated oxidized lipid species and red fluorescence indicated unoxidized lipid species. Lipid peroxidation is expressed as a ratio between green and red fluorescence sample signal multiplied by 100.

### Animal studies

All procedures involving animals were approved by the Institutional Animal Care and Use Committee of University of Texas Southwestern Medical Center. Eighteen Male, Sprague-Dawley rats, 7 weeks of age, were included in this study. All rats were housed in a temperature-controlled animal room (22± 2°C) under a 12h dark/light cycle, with access to laboratory food and water ad libitum (n=3 rats/plastic cage).

### Tumor cell inoculation

At the time of tumor cell inoculation rats were anesthetized with 2% isoflurane and a midline laparotomy incision (2cm) was made to expose the liver. N1S1 rat Hepatoma cells (1 x 10 (7)) in a matrigel suspension was injected into the lower left lobe of the liver. Eight days post implantation tumor growth was monitored using Magnetic resonance image (MRI). Studies were initiated once the tumors reached a diameter of 1.0 - 1.5 cm (approximately 10-12 days post tumor inoculation).

### Surgical placement of indwelling hepatic arterial infusion port

Placement of indwelling hepatic arterial infusion port were performed in tumor bearing rats. Anesthetized rats were placed in supine position, and a midline incision (~5 cm) was made to enter the peritoneal cavity. Under a surgical microscope, the hepatic artery was exposed and ligated distal to gastroduodenal artery (GDA) with a 6-0 silk tie. A small arteriotomy was made on GDA and the polyurethane intravascular 2Fr tubing tip end (inner diameter 0.3mm, outer diameter 0.6mm; Access Technologies, Skokie, Illinois, USA) of the implantable infusion port (silastic PMIN port; Access Technologies, Skokie, Illinois, USA) was inserted at the incision point on the GDA and advanced to the proper hepatic artery. The catheter was then secured by ligatures around the artery. Next a 1-2 cm pocket was created between the skin and muscle layer near the lower part of the abdominal incision to accommodate the implantable infusion port and the connected intra-arterial catheter. The implanted port was sutured and secured to the muscular fascia, followed by closure of the abdominal cavity using standard suture techniques. Thereafter, Taurolidine-Citrate Catheter lock Solution (TCS) (Access Technologies, Skokie, Illinois, USA) was injected into the port to ensure patency and lock the catheter. For the repeated hepatic artery infusion studies, LDL nanoparticles were injected percutaneously through the port, using a 24 gauge Huber point needle (Access Technologies, Skokie, Illinois, USA), followed by a TCS flush.

### Repeated hepatic arterial infusions

Rats were randomly allocated into three groups: group I, untreated control rats (n=9); group II, LDL-TO controls (n=4) and group III, LDL-DHA (n=10). Repeated treatments of LDL nanoparticles was performed through the hepatic arterial infusion port at baseline and days 3 and 6. The repeated treatments of LDL-DHA was administered at a dose of 2 mg/kg (DHA) each. LDL-TO treatments were given at an equivalent dose to LDL-DHA. The dose and treatment frequency was selected as a 2mg/kg dose was previous demonstrated to be an effective therapeutic dose for treating tumors and a 3 day dosing frequency was selected as therapeutic responses to HAI of LDL-DHA nanoparticles are near complete in this time frame. Over the course of study the animals’ body weights were monitored. All rats were sacrificed on the day 9 at which point blood and various organs were collected for histopathology and biochemical analyses.

### Magnetic resonance imaging (MRI)

MRI was performed on all rats at baseline, 3, 6, and 9 days post treatment. A 9.4T MR imaging system (Varian/Agilent, Santa Clara, USA) was used to acquire images with the following parameters: T2: TR/TEesp, 2500/10ms; echo-train length, 8; and T1: TR/TE 250/1.98ms, flip angle 70; the rest parameters of FOV, 64 × 64mm2; matrix size, 256 × 256; 18 slices without gap; section thickness= 2mm, averages= 6. For contrast-enhanced T1 weighted MRI, Magnevist (Bayer Schering Pharma AG, Berlin, Germany) was injected *via* the tail vein at a dose of 100 μL.

### Measurements of tumor volume, necrosis ratio and tumor volume doubling time (DT)

N1S1 Tumors appeared hypointense on T1 weighted images, and hyperintense on T2 and contrast-enhanced T1-weighted MR images. ImageJ software (National Institutes of Health, Bethesda, MD, USA) was used to measure the tumor size and necrosis ratio. Tumor area was measured on T1-weighted MR image, by manually circle the tumor lesion on all involved images with T2 image as reference. Tumor volume was calculated using the equation: tumor volume = Σ (tumor area on each slice × slice thickness).

The degree of treatment induced tumor necrosis was measured from the contrast enhanced T1-weighted images. Successful transarterial treatment typically display radiographic features of a non-enhancing core region surrounded by a thin rim enhancement indicative of central tumor necrosis accompanied with a periphery of enhancing cells. The area of central non-enhancing tissue was delineated on ImageJ to estimate necrosis. The necrosis ratio was defined as the volume of tumor necrosis over that of the entire tumor volume, i.e. necrosis ratio = Σ (area of necrosis × slice thickness)/(area of whole tumor × slice thickness) × 100%. Necrosis ratio obtained from MRI was further validated with the corresponding histopathology findings.

### Histopathological analysis

At the time of euthanasia, partial excised liver, tumor, and spleen samples were collected, and were fixed with total immersion in 10% neutral buffered formalin for 24h, then embedded in paraffin, and sectioned into 5-μm slices and stained with Hematoxylin and Eosin (H&E). The slides were captured with an optical microscope (CX31, Olympus, Japan) at 100× magnification for histopathology, and micrograph were taken with a microscope digital camera system (DP50, Olympus, Japan). Ki-67 immunohistochemistry was also performed on tissue sections using microwave antigen retrieval and Ki-67 antibody (1:100 dilution; Thermo Fisher Cat # MA5-14520). Tumor tissues from untreated and LDL-TO treated tumor bearing rats serves as controls.

### Necrosis ratio determination

H&E slides of control and treated tumors were scanned using a histology slide scanner (PrimeHisto XE) and the images imported into ImageJ. The approximate area of the tumor was marked and measured. The image was then split into Red, Green and Blue Channels. The viable part of the tissue was most distinctive in the red channel. An intensity threshold was applied until only the viable area was selected. These areas were then selected with the Wand tool and measured. The necrosis tissue ratio was computed for each of the slides percentage necrotic index expressed as (necrotic area)/(total tissue area) × 100.

### Serum collection and analysis

Immediately following euthanization, 5mL of blood was collected from inferior vena cava in all rats, and was centrifuged at 2500 rpm for 10 min at 4°C. Separated serum was collected into eppendorf tubes and stored at -20°C until analysis. Plasma liver enzymes alanine aminotransferase (ALT), aspartate aminotransferase (AST), gamma glutamyl transferase (GGT), total bilirubin (TBIL), albumin (ALB), glucose (GLU), alkaline phosphatase (ALKP), Triacylglyceride (TRIG), direct high density lipoprotein (dHDL), Cholesterol (CHOL), creatinine (CREA) and blood urea nitrogen (BUN) were measured with a AU400e automated biochemical analyzer.

### Western blot analysis

Frozen samples liver or tumor tissue (~40mg) were homogenized in 500μL Cell Lysis Buffer (Cell signaling technology 9803) with EDTA-free Protease Inhibitor Cocktail (Roche) while on the ice. Samples were then centrifuged for 10 minutes at 14,000 rpm at 4°C, and the collected supernatants were stored at −80°C Protein concentrations were measured by Pierce BCA Protein Assay Kit (prod#23227). Protein concentrations of the collected supernatants were determined using the Pierce BCA Protein Assay Kit (prod#23227). Then, equal amounts of sample (30 μg/lane) were separated on 10% SDS-PAGE and transferred onto Immobilon-FL PVDF membrane (Millipore, USA). After blocking with 5% nonfat milk, Western blotting was performed with primary antibodies against nuclear factor-ĸβ (NFκB-p65) (SC-514451), interleukin-6 (IL-6) (SC-57315), C reactive protein (CRP) (SC-69770) and glutathione peroxidase 4 (GPX4) (sc-50497) (all of 1:1,000, Santa Cruz) at 4°C overnight, and then incubated with secondary antibodies (all of 1:2500) for 1 h at room temperature. β-Actin (sc-47778)(1:1,000, Santa Cruz) was used as a loading control. The protein bands were detected by ECL detection system and densitometry was quantified using ImageJ software (National Institutes of Health, MD).

### Lipid peroxidation analysis

Liver and tumor peroxidative damage was assessed by measuring the malondialdehyde (MDA) level in tissue sample using the TBARs assay as described previously (36). Results were expressed as μM MDA formed/mg protein of tissue.

### GSH/GSSG assay

Total soluble glutathione (GSH) and glutathione disulfide (GSSG) were measured in tissue homogenates using the enzymatic recycling method (37). Briefly, about 50mg of tissue were homogenized in 10 volumes of cold 5% metaphosphoric acid and 0.6% sulfosalicylic acid mixture. Protein was precipitated and the supernatant was used to determine GSH and GSSG. Results were expressed in µmoles per gram of tissue.

### GC-MS fatty acid analysis

The gas chromatography-mass spectrometry lipidomics analysis of fatty acids in the liver was adapted from the protocol by Val et al. (38). Refer to **Supplemental Material** for detailed description of methods.

### Immunohistochemistry

To examine the contribution of early caspase mediate processes to LDL-DHA tumor killing N1S1-tumor bearing rats receiving a single transarterial infusion of LDL-DHA was examined 3 days post treatment. Tumor tissue was excised and immunohistochemistry was performed as described previously using microwave antigen retrieval and cleaved caspase-3 (1:50 dilution; Abcam, Cambridge, MA). Tumor tissues from untreated tumor bearing rats serves as controls.

Cleaved caspase3 and Ki67 quantification were estimated using IHC by labelling Formalin-fixed, paraffin-embedded (FFPE) tissue sections with respective antibodies and counterstaining with Hematoxylin. The slides were then scanned using the Hamamatsu Nanozoom Scanner and images analyzed by counting percent positive stained cells for antibody compared to total cells in a field of view using ImageJ Cell Count feature.

### Statistical analysis

Data were expressed as the mean ± standard error. Analysis of variance (ANOVA) with Tukey’s multiple comparison *post hoc* testing was used for evaluation of differences between groups. Differences with a P value less than 0.05 was deemed statistical significance. All statistical analyses were performed using GraphPad Prism Version 5.0 (GraphPad Software, San Diego, CA).

## Results

### Nanoparticle characterization

Extensive physicochemical and morphological characterization of the LDL- nanoparticles has been reported previously by our group (31, 39). In the following study the LDL-DHA nanoparticles displayed an average hydrodynamic diameter of 30.1 ± 0.5 nm and a negative zeta potential of -23.4 ± 0.9 mV. These particles were monodispersed showing a mean polydispersity index = 0.26 ± 0.1. Each LDL nanoparticle carried an estimated 1,672 ± 219 molecules of DHA. The DHA concentration in the stock solution of LDL-DHA nanoparticles was 9.4 mM.

The LDL-TO (LDL reconstituted with triolein) nanoparticles displayed similar physicochemical characteristics of plasma LDL. LDL-TO maintained a size of 24.5 ± 0.9 nm and average polydispersity index measured 0.30 ± 0.02. The zeta potential for these particles was -6.6 ± 0.5 mV). According to previous preparation each LDL is typically loaded with over 300 molecules of TO (31).

### LDL-DHA nanoparticles effects on N1S1 Cells

**Figure 1:**
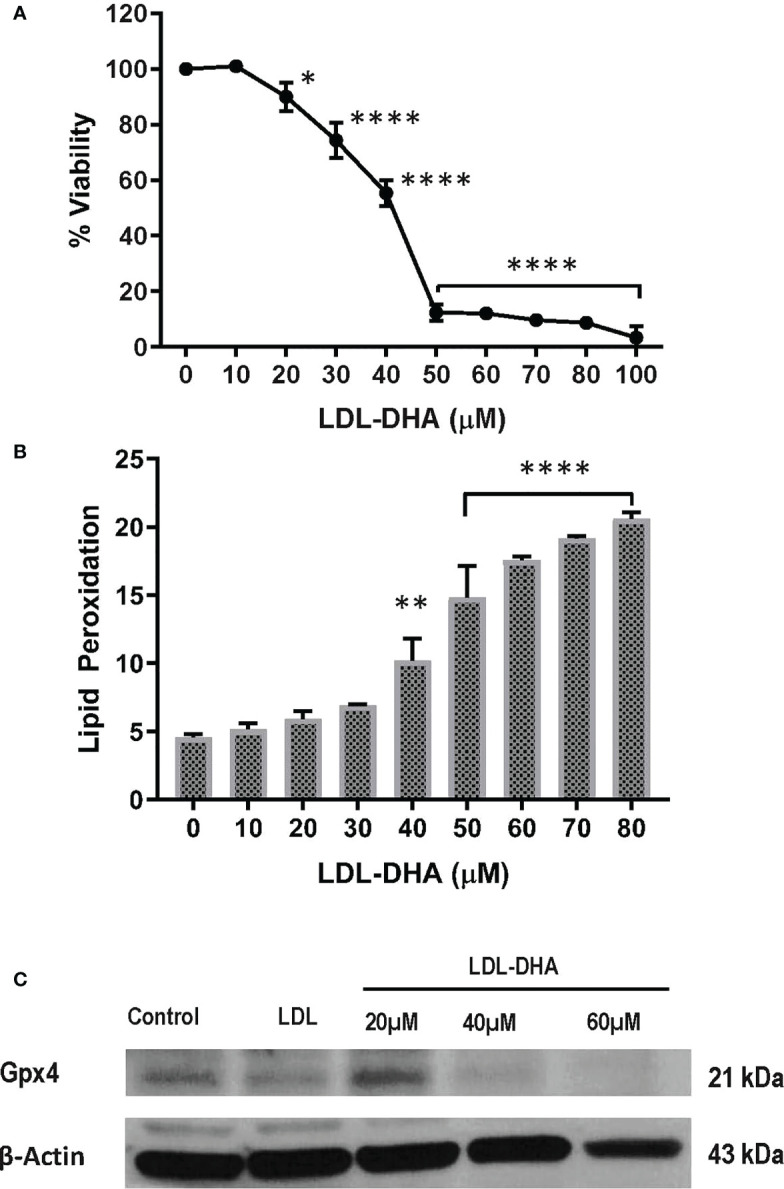
LDL-DHA induces toxicity on N1S1 cells through lipid peroxidation. **(A)** N1S1 cells were serum starved overnight, and then treated with LDL nanoparticle (0-100μM). Cell viability was measured by MTS/CCK assay at 72 hours after LDL nanoparticle treatment. Experiments were performed in triplicate wells with 3 independent runs. Results are expressed as mean ± SEM. *, P <0.05; ****, P <0.0001 compared with untreated control. **(B)** N1S1 cells were serum starved overnight, then treated with LDL nanoparticle (0-80µM) for 24 hours. Lipid ROS production was assessed by flow cytometry using C11-BODIPY. Results are expressed as mean ± SEM (n=3). **, P <0.01; ****, P<0.0001 compared with corresponding group (two-way ANOVA). Lipid ROS was not assessed over 80 µM due to inadequate amount of residual cells. **(C)** Immunoblot showing protein expression levels of GPX4 in untreated and treated N1S1 hepatoma cells 24hours after LDL (control) or LDL-DHA (20, 40 and 60 µM) exposure.

The cytotoxicity of LDL-DHA nanoparticles was evaluated in the N1S1 hepatoma cell line using a CCK-8 dose response assay. **Figure 1A** shows that the N1S1 cells were sensitive to the LDL-DHA nanoparticles from concentrations as low as 20µM. Thereafter, their viability dropped precipitously over the dose range from 20µM-50µM to menial levels. Over the second half of the dose response curve the viability of the N1S1 cells was gradually reduced to 0%. Lipid peroxidation was also measured over this dose range and lipid peroxides, as measured by Bodipy-C11 581/591 lipid, showed a dose dependent increase with increasing concentrations of LDL-DHA (**Figure 1B**). Accompanying this rise of lipid peroxidation was a concomitant depletion of the lipid antioxidant GPX4 (**Figure 1C**).

Comparative cell viability studies in response to increasing doses of LDL-DHA nanoparticles were performed on primary cultures of Sprague-Dawley rat hepatocytes. These experiments showed that LDL-DHA was not harmful to the primary rat hepatocytes over the dose range examined with the N1S1 cells (**Supplementary Figure 1**).

**Figure 2:**
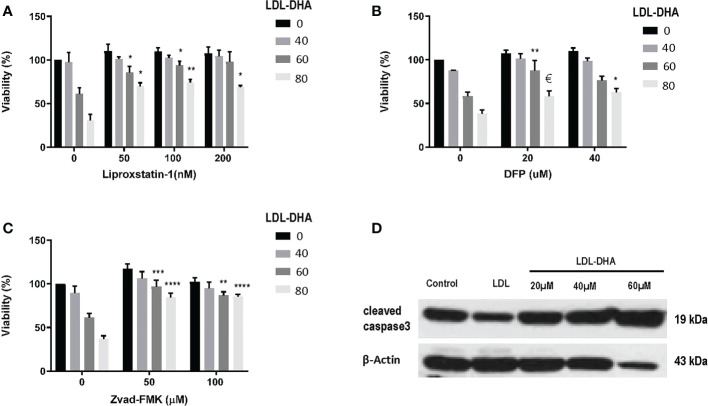
Identifying cell death pathways mediated by LDL-DHA cytotoxicity. Cells were treated with LDL-DHA (0, 40, 60, 80 μM) for 24 h in the absence/presence of: **(A)** liproxstatin (0, 50, 100, 200 μM); **(B)** DFO (0, 20, 40 μM); or **(C)** Zvad-FMK (0, 50, 100 μM). Cell viability (left panel) was measured by MTS assay at 24 hours after LDL-DHA treatment. Results are expressed as mean ± SEM (n=3). *, P <0.05; **, P <0.01; ***, P <0.001; ****, P<0.0001, €, P= 0.065 compared with corresponding LDL-DHA only treatment group. **(D)** Immunoblot of protein expression levels of cleaved caspase 3 in untreated and treated N1S1 hepatoma cells 24hours after LDL (control) or LDL-DHA (20, 40 and 60 µM) exposure.

Next the mechanism of LDL-DHA induced cytotoxicity was assessed in the N1S1 cells using specific inhibitors of cell death (**Figure 2**). The radical trapping antioxidant, liproxstatin-1 a potent inhibitor of ferroptosis, was able to significantly protect N1S1 cells from LDL-DHA’s cytotoxic effects (**Figure 2A**). The role of ferroptosis in LDL-DHA mediated tumor cell killing was further validated with the iron chelator, deferiprone (DFP), which was also able to block the cytotoxic activity of LDL-DHA (**Figure 2B**). In addition to the inhibition of ferroptosis, apoptosis inhibition was also investigated in our study. Surprisingly, ZVad-FMK, the pan-caspase inhibitor of apoptosis, was also shown to effectively rescue N1S1 cells from LDL-DHA killing (**Figure 2C**). These findings were further validated by increased expression of cleaved caspase-3 among LDL-DHA treated cells (**Figure 2D**). Collectively these findings indicate that LDL-DHA nanoparticles are able to induce both ferroptotic and apoptotic pathways of cell death in N1S1 hepatoma cells.

To demonstrate that the anticancer response to LDL-DHA was not a murine specific effect we evaluated the well differentiated human HCC cell line HUH7 (**Supplementary Figure 2**). These HCC cells were shown to have similar sensitivities to LDL-DHA as the N1S1 cells. Furthermore, select ferroptosis and apoptosis inhibition was shown to rescue cells from LDL-DHA cytotoxicity. Similarly, the later was supported by increased protein expression of cleaved caspase 3 following LDL-DHA treatments. Of note at higher concentrations of LDL-DHA treatment (> 40µM) cleaved caspase 3 levels decrease. This likely reflects a predominance of ferroptotic cell killing at these doses of LDL-DHA.

### Port placement

**Figure 3:**
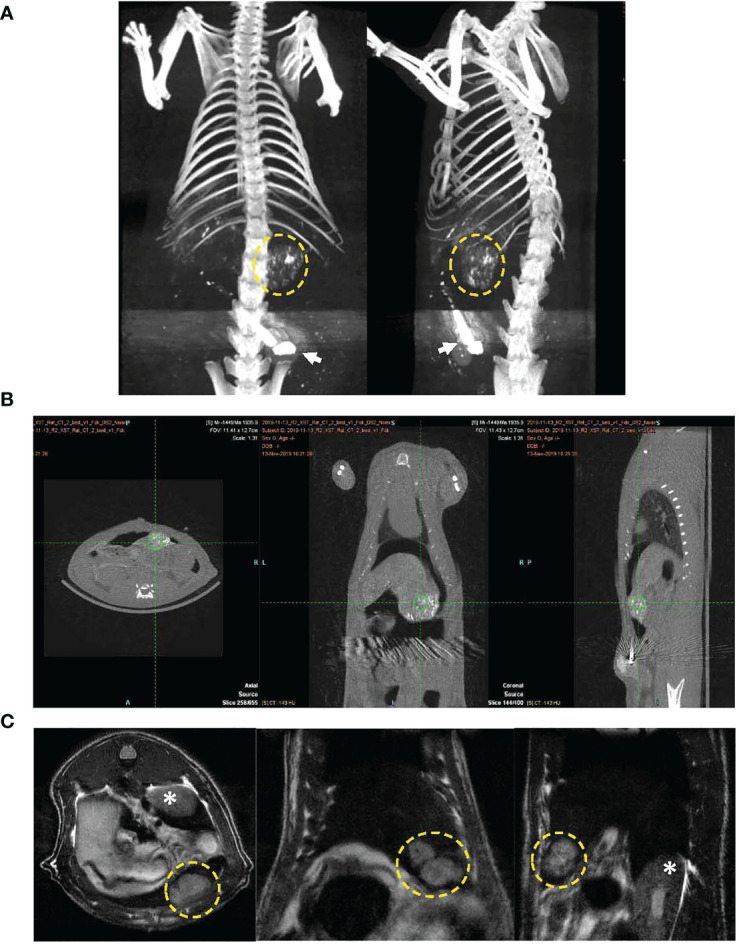
CT image of port-catheter system in the N1S1 tumor bearing rat. CT scan of the rat abdomen with three-dimensional reconstructed coronal image. The port system is filled with contrast material (Ethiodized oil) for visualization of the port (arrow) and the catheter line feeding into the gastroduodenal artery. Contrast material was also administered into the animal, some aspects of the liver’s contour, hepatic vasculature and tumor (dashed yellow circle) can be seen. **(B)** CT images in three planes showing soft tissue anatomy of tumor bearing rat. Accumulation of contrast material can be seen in the tumor (green hatch marks). **(C)** Multiplane T2 weighted MRI of N1S1 tumor bearing rat. Tumors appear hyper intense on images (dashed yellow circle). * indicates kidney.

MRI was performed approximately 10 days post tumor inoculation in each rat to assess growth of N1S1 tumors. Once tumors were radiologically confirmed rats underwent surgical placement of the arterial port catheter system. Micro CT imaging was performed in a cohort of rats to demonstrate successful surgical implantation of the port-catheter systems (see **Figures 3A, B**). Multiplane T2-weighted MRI also clearly shows of the orthotopic hepatoma as a hyper-intense lesion (**Figure 3C**). Corresponding contrast enhanced micro CT, post port placement, shows positioning of the port in the lower abdomen, catheter advancement into the hepatic artery and successful deposition and accumulation of contrast in the rat Hepatoma (**Figures 3A, B**). The liver is also delineated in this image as contrast also distributes throughout the hepatic arterial circulation (**Figure 3A**). Having demonstrated placement of the percutaneous port and successful perfusion of hepatomas with CT contrast experiments were initiated and hepatic arterial infusion of LDL nanoparticles commenced in study rats approximately 3 hours post port placement.

Overall, the port placement and repeated infusions were tolerated well by all animals over the course of the study. No observable clinical signs of weakness, restlessness, piloerection, tremors, hair loss or diarrhea appeared in in the study cohort over the 9-day infusion course and all animals survived until the study end-point. Over the course of the study the animals’ body weight was documented (see **Supplementary Figure 3**). Control rats experienced steady incremental increases in body weight throughout the study, while LDL-DHA and LDL-TO rats showed a 5 and 2% decrease, respectively, in weight in the early post-operative period. Thereafter their body weights gradually increased over the remainder of the study. No significance difference in the mean body weight was observed between the control, LDL-TO and LDL-DHA treated groups.

### Treatment results

**Figure 4:**
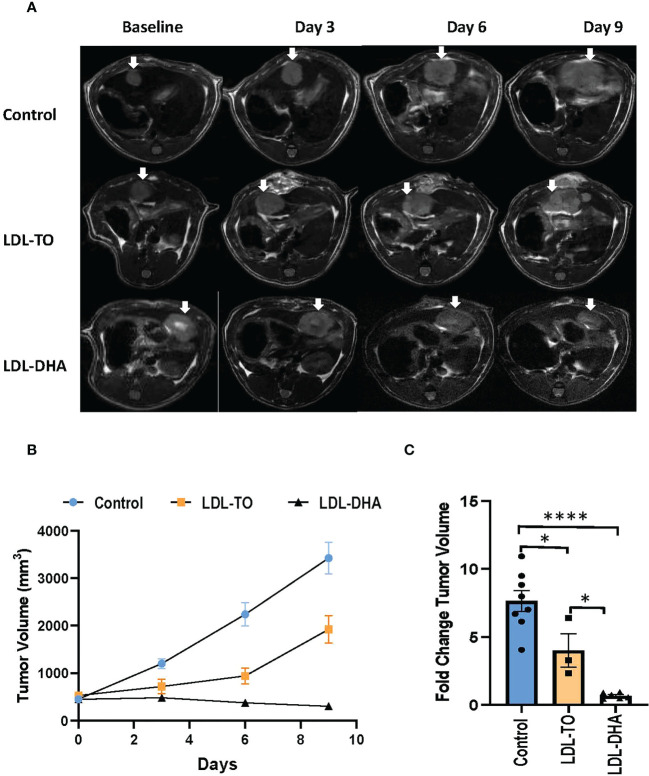
Tumor growth over course of repeated LDL nanoparticle treatments. **(A)** MRI assessment of tumor-bearing rats over the course of repeated LDL Nanoparticle treatments. Representative T2-weighted axial images of untreated controls, LDL-TO and LDL-DHA allocated rats at baseline, 3 days, 6 days and 9 days after LDL nanoparticle treatments. Tumor appear hyper-intense on T2-weighted images. White arrow indicates tumor. **(B)** N1S1 tumor volumetric assessment versus time over a course of repeated LDL nanoparticle treatments or no treatments. The average tumor volume for each group is presented in absolute volume (as calculated from MRI) and expressed as mean ± SEM. **(C)** Fold change in tumor volume at day 9 relative to corresponding baseline values. Significant differences between groups is expressed as mean ± SEM. *, P< 0.05; ****, P< 0.0001.

Noninvasive MRI was used to monitor tumor response following locoregional treatment over the nine day study period (**Figure 4A**). The margins of the hyperintense orthotopic N1S1 hepatoma can clearly be demarcated from the surrounding liver on the T2 weighted images. Over the course of the nine day study period the untreated tumor grows at an exponential rate (**Figures 4B, C**). At day 9 the volume of the untreated tumor is close to 9 times its original volume. The LDL-TO treated tumors continued to experience growth over the nine days, however, it was not as rapid as the untreated controls (4 times increase over baseline). The effects of repeated LDL-DHA treatment was evident as the tumor size actually regressed over the study period. At day 9 the tumor volume was less than that at baseline (Day 9/baseline volume ratio=0.83). Image-based growth curves clearly show the dynamics of the treatment effects over the study period.

**Figure 5:**
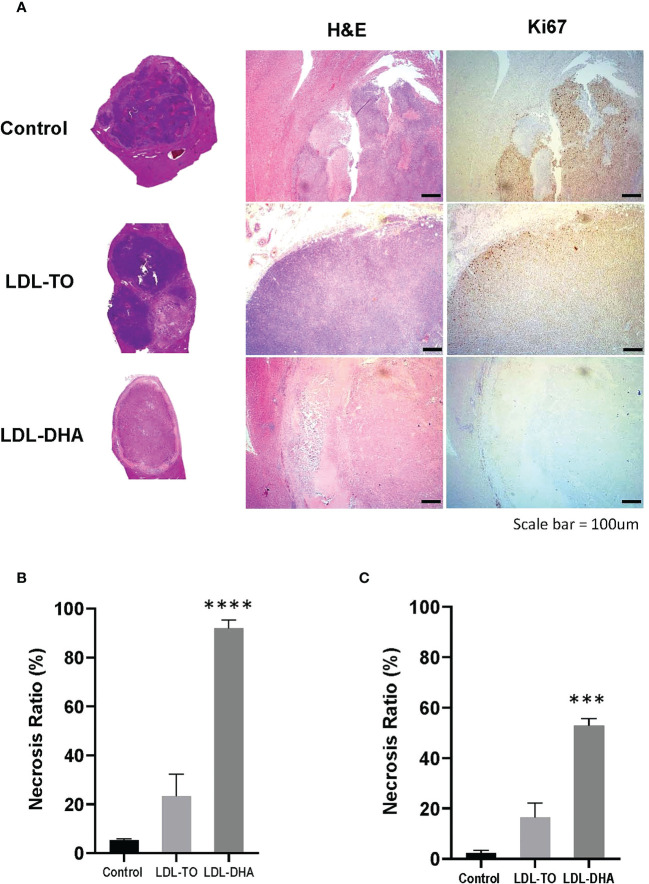
Tumor Viability following repeated LDL nanoparticle treatments. **(A)** Liver histology and immunohistochemistry of untreated controls and rats receiving repeated HAI of LDL nanoparticles. Gross tissue micrographs, hematoxylin and Eosin histological sections, corresponding IHC sections stained for stained for the proliferation marker Ki-67 and the apoptosis marker cleaved caspase-3. Images were taken at 20x magnification. Dotted line indicates liver tumor boundary. The LDL-TO sections are entirely composed of tumor tissue. Necrosis ratio measurements subfigure 5B and 5C. Necrosis ratio is defined as the volume of tumor necrosis over that of the entire tumor volume x 100%. **(B)** Necrosis estimates from histopathology. **(C)** Necrosis estimates from T1 weighted MRI. See *Methods* for details. Data is expressed as % mean ± SEM. ***, P<0.001; ****, P< 0.0001 versus untreated or LDL-TO controls.

Specimens taken at the termination of the study also showed drastic histological differences (**Figure 5A**). The untreated tumors consisted mainly of dark basophilic staining tissues indicative of highly viable and active tumor tissue. LDL-TO treated tumors also displayed large regions of viable tissue, this however, was accompanied with sparse regions of tumor necrosis. In contrast the LDL-DHA treated tumors were composed mainly necrotic non-viable tissue. Histologic estimates of necrosis measured as high as 92% for the LDL-DHA group, while Untreated controls and LDL-TO groups measured 5 and 23% respectively (**Figure 5B**). Radiologic measurements of necrosis also followed a similar trend with Controls, LDL-TO and LDL-DHA groups averaging 2, 16 and 53% necrosis respectively (**Figure 5C**).

Complementary Ki67 staining performed on histological specimens also displayed consistent results as the H&E findings (**Figure 5A**). Untreated control and LDL-TO treated tumor sections were replete with Ki67 positive staining cells, supporting high proliferative and active tumor tissue. Conversely, the LDL-DHA treated tumors were void of Ki-67 staining. This is consistent with the high estimates of tumor necrosis associated with LDL-DHA treatment. Quantification of Ki67 staining confirmed significantly lower levels of Ki67 positive cells within the LDL-DHA treated tumors (12.7 ± 5.2%) relative to untreated (58.5 ± 6.3%) and LDL-TO treated tumors (57.2 ± 1.6%) (**Supplementary Figure 4**).

### Serum biochemistry

The serum biochemistry values were mostly similar between the study groups (**Supplementary Table 1**). Higher levels of serum alkaline phosphatase were detected in LDL-DHA treated rats compared to the controls. While serum triglycerides were higher in LDL-TO treated rats relative to control and LDL-DHA treated groups. All other measurements of liver, renal and metabolic functions were unremarkable across the groups.

### Inflammatory regulators

**Figure 6:**
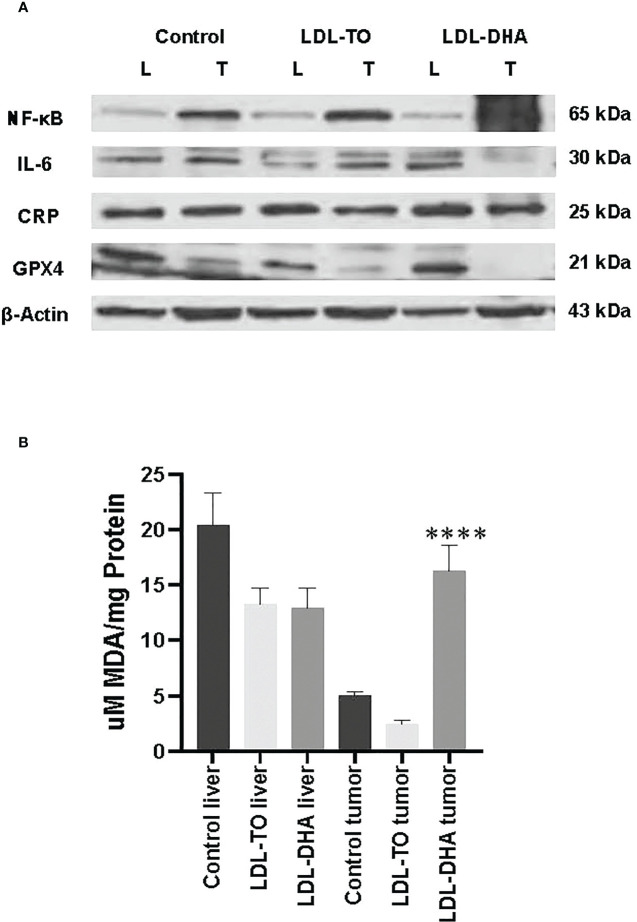
Inflammation and lipid peroxidation markers in LDL nanoparticle treated tumors. **(A)** Protein expression levels of inflammatory response marker and glutathione-peroxidase 4. Protein expression of hepatic nuclear factor-ĸβ (NF-ĸβ), interleukin-6 (IL-6) C reactive protein (CRP) and glutathione peroxidase 4(GPX4) in liver and tumor samples from untreated controls and rats with repeated HAI of LDL nanoparticles. Representative blots are shown. The data is shown relative to β-actin expression for each treatment group. **(B)** Levels of lipid peroxidation in liver and tumor samples from untreated controls and rats following repeated HAI of LDL nanoparticles. The TBARS assay measured lipid peroxide levels per mg of tissue. The data are expressed as µM malondialdehyde (MDA) per mg of tissue protein (mean ± SEM) for each treatment group **(B)**. ****, P < 0.0001 versus untreated and LDL-TO groups.

Western blot analyses were performed to assess the liver and corresponding tumor expression of inflammatory regulators, NF-κB, IL-6 and CRP, across the study groups (**Figure 6A**). The expression levels of the master regulator, NF-κβ, remained constant in the liver regardless of intervention. However, within the tumor the levels of NF-κβ increased dramatically over the untreated and LDL-TO treated groups. Liver CRP expression moderately increased with LDL-DHA treatment over the corresponding controls (1.5 fold). Within the tumor CRP levels remained constant regardless of treatment. Lastly, with regards to IL-6, untreated and LDL-DHA treated livers had similar expression, however LDL-TO treated liver experience a marked decrease in this immune modulator. On the other hand, tumor IL-6 levels were similar for control and the LDL-TO groups, while the LDL-DHA treatment resulted in a precipitous drop in IL-6.

### Metabolic evaluation

#### GPX4 protein expression

We also extended the use of Western blot analyses to investigate tissue levels of the antioxidant GPX4 (**Figure 6A**). Equivalent expression of GPX4 was detected for control and LDL-DHA treated livers, but moderately lower levels were seen in the livers following LDL-TO infusions. Hepatoma tissues also showed slightly lower levels of GPX4 in LDL-TO group compared to untreated controls, but the LDL-DHA treatment group experienced an even greater diminution of GPX4.

#### Tissue glutathione

In keeping with the GPX4 response, tissue measurements glutathione revealed that the reduced form of this metabolite (GSH) did not differ in the livers from the different groups (**Supplementary Figure 5**). Tumor GSH remained at similar levels between control and LDL-TO treatments. LDL-DHA infusion resulted in a greater than 3 fold reduction in tumor GSH, however this decrease did not reach statistical significance. The oxidized glutathione metabolite (GSSG) remained constant regardless of tissue or treatment regime.

#### Tissue malondialdehyde

The levels of MDA were used to assess the extent of lipid peroxidation experienced by liver and tumor from each study group (**Figure 6B**). Throughout the study levels of lipid derived aldehyde varied little between untreated and LDL nanoparticle treated livers. Conversely, within the tumor LDL-DHA treatment elicited pronounced increase in lipid peroxidation. MDA concentrations in LDL-DHA treated tumors were approximately 3 and 8 x greater than that in untreated and LDL-TO treated counterparts.

### Lipidomics

Lipidomics was employed to assess alteration in lipid composition and metabolism following HAI of LDL nanoparticles. Organic extraction methods enabled GC-MS investigation of polar (phospholipid) and neutral (triglycerides, cholesterol-ester) lipid fractions (**Supplementary Tables 2**-**5**). The composition of the polar lipids in the liver and tumor did not differ significantly between the cohorts of untreated, LDL-TO or LDL-DHA treated rats. Conversely, amongst the neutral lipids the levels of FA(20:2n-6) were elevated in the liver following LDL-DHA treatment relative to untreated and LDL-TO treated livers. For the tumor samples HAI of LDL-DHA lead to accumulation of saturated, mono-and polyunsaturated FAs (16:0, 18:1n-7, 20:4n-6, 20:3n-3, 20:2n-6, 22:6n-3, 22:5n-3) within the neutral lipid pool compared to their untreated and LDL-TO counterparts.

### Cleaved caspase-3 immunohistochemistry

To assess the mechanistic contributions of caspase-mediated processes to LDL-DHA induced tumoricidal effects time points earlier than 10 days post treatment seen in these studies was required. Tumor samples from N1S1 tumor-bearing rats 3 days following a single HAI of LDL-DHA was selected for this study. Tumors showing a partial response with residual viable tumor was examined for cleaved caspase-3 expression (**Supplementary Figure 6**). Positive staining for cleaved caspase-3 was detected throughout this section, suggesting that caspase mediated processes are active earlier in the course the LDL-DHA tumor cell killing. Caspase-3 expression was also detected in untreated controls but this was only found around regions of spontaneous necrosis. Quantification of Cleaved caspase-3 positive cells revealed overall similar levels between untreated controls (7.5 ± 1.0%) and LDL-DHA treated tumors (5.1 ± 0.3%) (**Supplementary Figure 6**).

## Discussion

Percutaneous implanted port-catheter systems for intra-arterial infusions hold promise for a growing number of applications in clinical oncology. Repeated hepatic arterial delivery of anticancer agents to the liver by implanted port-catheter systems has been described for the treatment of advanced unresectable liver cancer (40, 41). Even among HCC patients with marked underlying liver disease this approach has been proven efficacious (11). Hepatic arterial infusions provide the benefits of “first pass and increased local concentration effects” within the tumor due to the preferential arterialization of hepatic neoplasms (3). Despite these pharmacokinetic advantages current transarterial therapies remain palliative (42). As such, the five year survival rate for HCC remains less than 12% (43). Innovative and novel therapeutic agents are urgently sought to address this unmet need. Preclinical large animal models, such as pigs, have been avidly used to demonstrate the feasibility of intra-arterial port catheter systems (44, 45), however, laboratory rodents are widely recognized as the primary animal model systems for oncology research. Implanting percutaneous intra-arterial port-catheter systems in rodents is a surgically challenging task, due to their small anatomy, but in recent years several groups have reported successful placement of hepatic artery port-catheter systems in the rat for evaluations of experimental anti-HCC therapies (21–23). Studies from our own group have also demonstrated the utility of hepatic artery port catheter systems in the rat (46). We assessed the safety of repeated transarterial infusions of LDL-DHA to the normal rat liver. Repeated infusions of LDL-DHA nanoparticles did not elicit hepatotoxicities and was concluded to be safe (46). Precedence for investigating the anticancer benefits of natural n-3 PUFAs stem from the seminal work of Sawada and others who reported that dietary intake of n-3 PUFAs among populations with chronic hepatitis significantly reduced the risk of HCC development (24). Other reports have also confirmed these findings (47). Within our own group formulation of DHA into LDL carriers has been shown to effectively kill hepatoma cells in culture by the induction of ferroptosis (33). Similar antitumor effects were also witnessed *in vivo* either by direct tumor injection or by transarterial injection (33, 34). In the present study, we sought to determine whether repeated infusions of LDL-DHA nanoparticles, through an indwelling cannula surgically implanted in the hepatic artery, would provide sustained tumor suppression in a syngeneic rat model of HCC.

Our investigations first examined the anticancer effects of LDL-DHA nanoparticles on *in vitro* cultures of the rat N1S1 and human HUH7 hepatoma cells. Similar to previous studies the LDL-DHA nanoparticles displayed a dose dependent cytotoxicity against the N1S1 and HUH7 cells, while primary Sprague Dawley rat hepatocytes remained fully viable over a similar treatment range. Accompanying the tumor cell cytotoxicity was a dose dependent accumulation of lipid peroxides. This finding (along with the depletion of GPX4 expression) point to ferroptosis as the mechanism whereby LDL-DHA induces tumor cell death. Rescue experiments with the radical-trapping antioxidant, liproxstatin-1 or ferrostatin, further confirmed the role of ferroptosis in the LDL-DHA’s cytotoxicity. Surprisingly, the apoptosis inhibitor, Z-vad-fmk, was also able to effectively protect the tumor cells from LDL-DHA killing. These findings of dual ferroptosis–apoptosis tumor cell killing was also observed in human HUH7 HCC cells following treatment with LDL-DHA. Collectively, these findings suggest that the LDL-DHA nanoparticles induce a mixed pattern of tumor cell killing which involve both ferroptotic and apoptotic pathways. Similar responses were also found *in vivo* and will be discussed later.

Selective tumoricidal activity against N1S1 hepatoma of was also observed following repeated transarterial infusions of LDL-DHA nanoparticles. Both radiologic and histological analyses confirmed striking therapeutic effects from the locoregional nanoparticle treatment on the rat tumors. Although some discrepancy was noted between the two analyses. While contrast-enhanced MRI is used to determine viable tumor tissue (enhancing portions of the tumor are presumed to be viable, whereas non-perfused portions are presumed to be necrotic) (48), it is unable to distinguish viable cells from permeable reactive granulation tissue (49). Hence, enhancing granulation tissue remains a potential confounding factor for radiologic-based measures of tumor necrosis. Contrast enhancing granulation tissue is often noted at the periphery of tumors following locoregional treatment (50) and thus likely explains the lower MRI measures of tumor necrosis in the present study. Histological analysis, the gold standard of treatment assessment, revealed near complete necrosis (~92%) of the hepatomas after 3 courses of the LDL-DHA nanoparticle treatment. Complementary immunohistochemistry also supported these measurements as Ki-67 staining was near absent in the LDL-DHA treated tumors (12.7 ± 5.2%), but abundant in corresponding untreated (58.5 ± 6.3%) and LDL-TO controls (57.2 ± 1.6%). Tumor volumes measurements also corroborated these histology findings as the LDL-DHA treated tumors did not show signs of growth over the study. In fact, the volume of LDL-DHA treated tumors decreased in this study suggesting evidence of treatment induced tumor regression. Conversely, the untreated cohort of untreated rat showed exponential tumor growth. The control LDL-TO treated group also displayed tumors growth, although it was at a delayed rate. Tumors from these animals presented varied degrees of necrosis (23.4 ± 9.0%) which were significantly greater than that seen in the untreated group (5.2 ± 0.7%). Given that the LDL-TO nanoparticles are known to support and not impede tumor growth these results revealed that other confounding factors were at play (34, 51). Positioning of the distal end of the fixed catheter that advances from the gastroduodenal artery into the hepatic artery proper could potentially obstruct arterial blood flow to the liver. The outer diameter of the catheter is 0.6 mm while that of the hepatic artery in the rat is 0.2 - 0.5 mm (52). Such impediment in hepatic artery blood flow would potentiate ischemic injury to the N1S1 liver tumors. As such, the LDL-TO treated tumors experienced some damage in the early post op period following port implantation, but soon after that the tumor growth resumed reaching exponential rates towards the end of the study. It stands to reason that the LDL-DHA treated tumors also experienced some obstructed hepatic arterial flow and ischemic injury as a result of the implanted port, however, the repeated administration of LDL-DHA nanoparticles appear to predominantly account for the observed tumoricidal effect. The biochemical and molecular disturbances that characterized the LDL-DHA treated tumors were absent in the tumors from the untreated and LDL-TO treated groups. Striking depletions in GSH and GPX4 accompanied by increasing levels of lipid peroxidation showcase the hallmark features of LDL-DHA induced ferroptosis (53). These tumors also significantly overexpressed NF-ƙβ indicating marked inflammatory activity following LDL-DHA treatment. We observed a significant accumulation of mononuclear inflammatory cells within the rim of the necrotic tumor mass after repeated LDL-DHA administration. Innate immune responses, including the recruitment and activation of mononuclear cells of the phagocytic system, likely contribute to the enhanced tumoricidal effects observed in this study.

The selective cytotoxic effects of LDL-DHA is also evident in the altered neutral lipid profile of treated tumors. The increased tissue levels of saturated, mono- and polyunsaturated FA moieties of neutral lipids suggest the intracellular accumulation of lipid droplets. Tumor accumulation of lipid droplets has been widely reported to correlate with chemotherapy treatments (54). Apoptosis-inducing anticancer drugs, like etoposide and doxorubicin, have been shown cause lipid droplet accumulation in tumor cells likely through the activation of p53 and the inhibition of mTOR and MYC which leads to lipid accretion as a result of mitochondrial impairment, inhibition of fatty acid oxidation and the subsequent redirection of fatty acids towards lipid storage (55, 56). Increased lipid droplet formation during chemotherapy may also be a consequence of non-apoptotic cell death pathways, as ferroptosis-induced cell death is known to perturb FA metabolism (57). Regardless of the mechanism of cytotoxicity stressed tumor cells respond by accumulating fatty acids and depositing lipid droplets (58). Similar to other anticancer agents LDL-DHA nanoparticle tumor cell killing is also characterized by the accumulation of neutral lipids. Further studies will be designed to elucidate how neutral lipid accumulation assist in mediating LDL-DHA induced tumor cell death.

Consistent with the *in vitro* findings the N1S1 tumors also displayed signs of caspase activated pathways/apoptosis following LDL-DHA treatments. In recent years several small molecules and natural products have also demonstrated the capacity to induce multiple pathways of cell death (59–61). Seiler and others have reported that the depletion of GPX4 is not only a potent activator of ferroptosis but may also be involved in potentiating apoptosis (62). Other potential mechanisms for co-stimulatory cell death pathways include the induction of pro-apoptotic protein PUMA by ferroptotic agents (59). The unique mixed pattern of LDL-DHA induced ferroptosis and apoptosis is intriguing and certainly requires further mechanistic exploration.

In conclusion, the percutaneous placement of a port-catheter system for repeated drug infusion is technically safe and provides a viable approach for accessing the hepatic artery. However, for laboratory small animal applications, smaller diameter catheters are recommended to prevent any complications of obstructive arterial blood flow. In spite of this mishap, repeated infusions of LDL-DHA proved to be highly effective at delivering DHA to rat hepatomas. The tumor deposited DHA was able to induce pronounce destruction of tumor tissue *via* ferroptosis and apoptotic pathways. Collectively, the results from this study demonstrates that repeated administrations of LDL-DHA nanoparticles through the hepatic artery provides sustained repression of malignant tumors in the rat.

## Data availability statement

The original contributions presented in the study are included in the article/**Supplementary Material**. Further inquiries can be directed to the corresponding author.

## Ethics statement

The animal study was reviewed and approved by Institutional Animal Care and Use Committee, UT Southwestern Medical Center.

## Author contributions

YW: Acquisition and analysis of data; study design; draft article; JL: Acquisition and analysis of data; study design; draft article; GD: Acquisition and analysis of data; critical revision of content; JC: Acquisition and analysis of data; draft article; critical revision of content; AA: Acquisition of data; critical revision of content; JM: Data curation, formal analysis, methodology; TQ: Contribution and support for the study; HZ: Contribution and support for the study; IC: Contribution to conception and design; Analysis and interpretation of data; Draft article and critical revision; Final approval for publication; Accountable for all aspects of work. All authors contributed to the article and approved the submitted version.

## Funding

This work was supported in part by Remeditex ventures LCC OTD-109946; NCI, National Institutes of Health (NIH), Grant R01CA215702; Provincial Ministry Co-Construction Key Project, Medical Scientific and Technological Research Program of Henan Province (SBGJ202002012); NIH HL020948 and the UTSW Cancer Center Support Grant (5P30 CA 142543-05).

## Conflict of interest

The authors declare that the research was conducted in the absence of any commercial or financial relationships that could be construed as a potential conflict of interest.

## Publisher’s note

All claims expressed in this article are solely those of the authors and do not necessarily represent those of their affiliated organizations, or those of the publisher, the editors and the reviewers. Any product that may be evaluated in this article, or claim that may be made by its manufacturer, is not guaranteed or endorsed by the publisher.

## Supplementary material

Supplementary Figure 1 Effects of LDL nanoparticles on Sprague Dawley rat primary hepatocytes. **(A)** MTS dose response assay of rat primary hepatocytes to LDL-DHA (0-100 µM). Experiments were performed in triplicate wells with six independent runs. Results are expressed as mean ± SEM. ***, P<0.001 versus untreated control.

Supplementary Figure 2 Identifying cell death pathways in human HUH7 cells mediated by LDL-DHA cytotoxicity. **(A)** HUH7 cells were serum starved overnight, and then treated with LDL nanoparticle (0-100μM). Cell viability was measured by MTS/CCK assay at 72 hours after LDL nanoparticle treatment. Experiments were performed in triplicate wells with 3 independent runs. Cells were treated with LDL-DHA (0, 40, 60, 80 μM) for 24 h in the absence/presence of: **(B)** ferrostatin (0, 20, 40 μM); **(C)** Zvad-FMK (0, 50, 100 μM). Cell viability (left panel) was measured by MTS assay at 24 hours after LDL-DHA treatment. Results are expressed as mean ± SEM (n=3). *, P <0.05; **, P <0.01; ***, P <0.001; ****, P<0.0001, Ψ, P= 0.07-0.08 compared with corresponding LDL-DHA only treatment group. **(D)** Immunoblot of protein expression levels of cleaved caspase 3 in untreated and treated HUH7 hepatoma cells 24hours after LDL (control) or LDL-DHA (20, 40 and 60 µM) exposure.

Supplementary Figure 3 Mean body weight of tumor bearing rats with implanted hepatic artery port-catheters receiving repeated infusions of saline or LDL nanoparticles over a 9 day study period. Arrows indicate time of HAI.

Supplementary Figure 4 Quantification of KI-67 immunohistochemistry in untreated controls, LDL-TO and LDL-DHA treated animals. Results are expressed as mean ± SEM.

Supplementary Figure 5 Concentrations of reduced and oxidized glutathione in liver and tumor samples from untreated controls and rats following repeated HAI of LDL nanoparticles. The data is expressed as µmoles of glutathione per mg of tissue (mean ± SEM) for each treatment group.

Supplementary Figure 6 H&E and Immunohistochemistry of N1S1 tumors after Sham and HAI treatment of LDL-DHA (2 mg/kg). N1S1 tumor tissue samples were collected 72 hours post-treatment. Corresponding tissue sections were stained for H&E and the apoptosis marker cleaved caspase-3. **(A)** Sham; **(B)** LDL-DHA. Images were taken at 20x magnification. Quantification of cleaved caspase-3 immunohistochemistry in untreated controls and LDL-DHA treated animals. Results are expressed as mean ± SEM.
