# Supplementary figures and images for: Repeated trans-arterial treatments of LDL-DHA nanoparticles induce multiple pathways of tumor cell death in hepatocellular carcinoma bearing rats
Source: Front Oncol. 2022 Nov 24;12:1052221. doi: 10.3389/fonc.2022.1052221 (PMC9730405; doi:10.3389/fonc.2022.1052221)

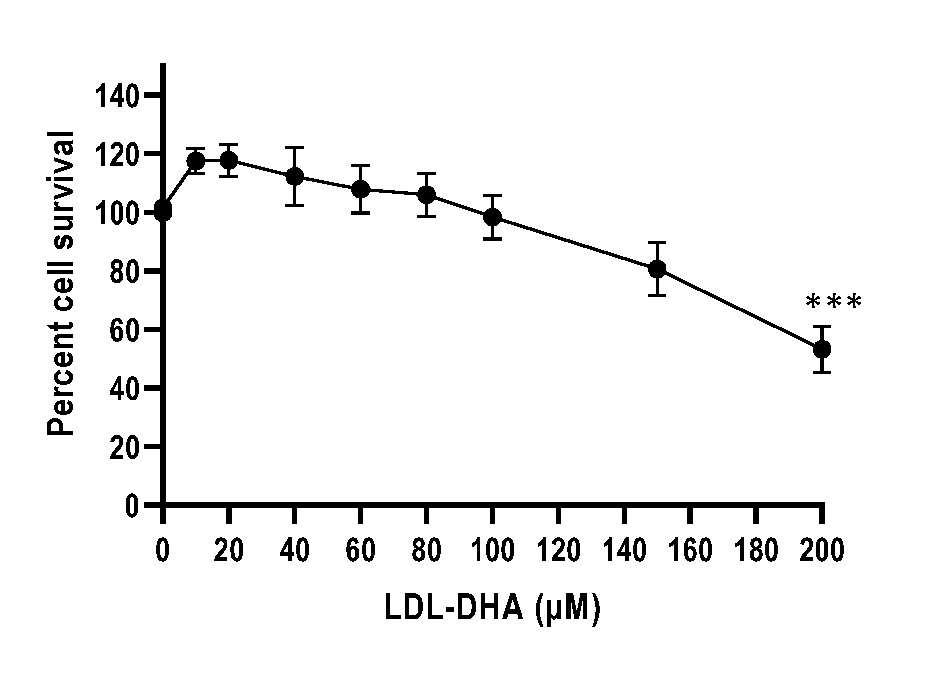

Supplement: Supplementary Figure 1 — Effects of LDL nanoparticles on Sprague Dawley rat primary hepatocytes. (A) MTS dose response assay of rat primary hepatocytes to LDL-DHA (0-100 µM). Experiments were performed in triplicate wells with six independent runs. Results are expressed as mean ± SEM. ***, P<0.001 versus untreated control. [file Image_1.jpeg]

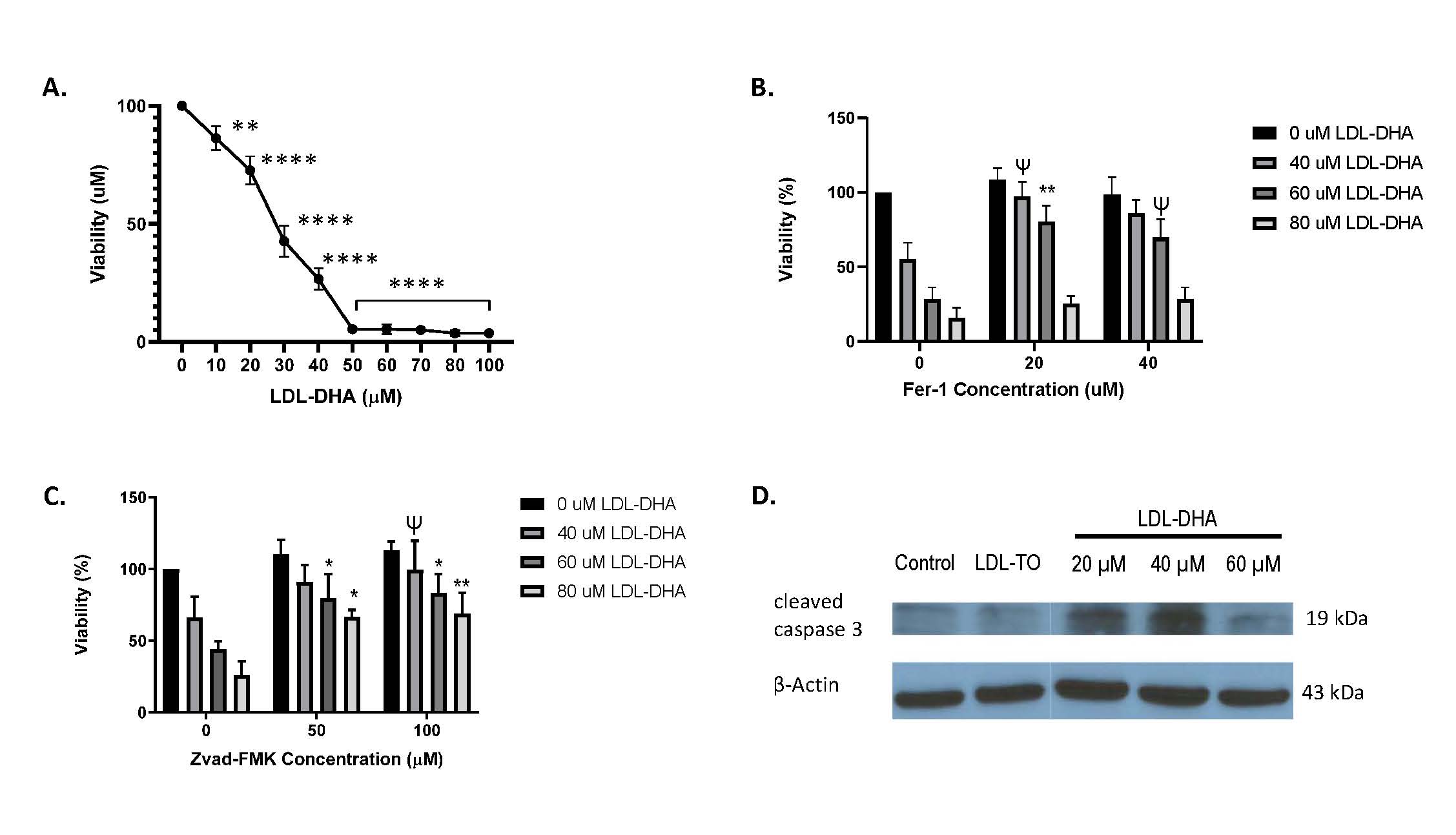

Supplement: Supplementary Figure 2 — Identifying cell death pathways in human HUH7 cells mediated by LDL-DHA cytotoxicity. (A) HUH7 cells were serum starved overnight, and then treated with LDL nanoparticle (0-100μM). Cell viability was measured by MTS/CCK assay at 72 hours after LDL nanoparticle treatment. Experiments were performed in triplicate wells with 3 independent runs. Cells were treated with LDL-DHA (0, 40, 60, 80 μM) for 24 h in the absence/presence of: (B) ferrostatin (0, 20, 40 μM); (C) Zvad-FMK (0, 50, 100 μM). Cell viability (left panel) was measured by MTS assay at 24 hours after LDL-DHA treatment. Results are expressed as mean ± SEM (n=3). *, P <0.05; **, P <0.01; ***, P <0.001; ****, P<0.0001, Ψ, P= 0.07-0.08 compared with corresponding LDL-DHA only treatment group. (D) Immunoblot of protein expression levels of cleaved caspase 3 in untreated and treated HUH7 hepatoma cells 24hours after LDL (control) or LDL-DHA (20, 40 and 60 µM) exposure. [file Image_2.jpeg]

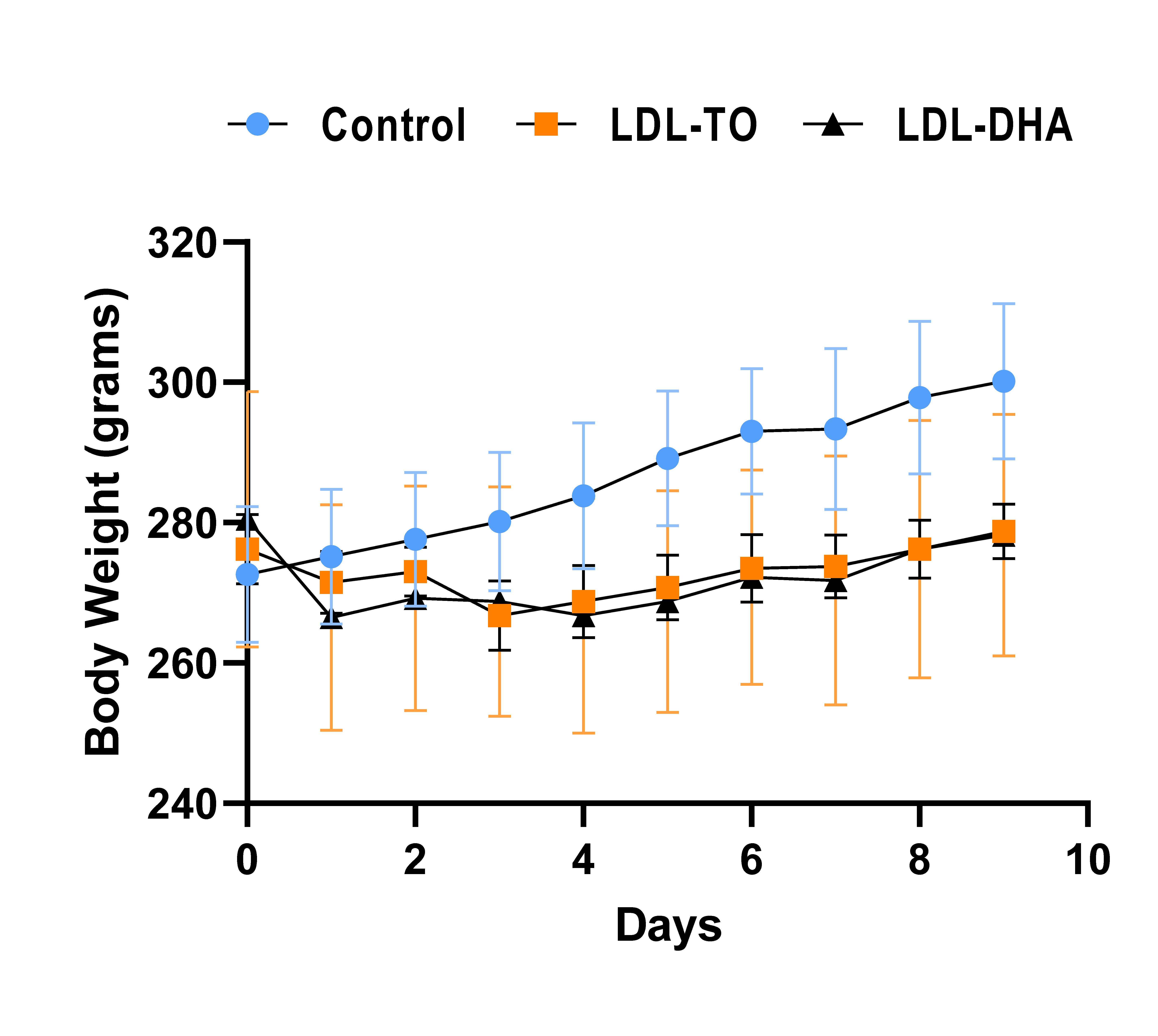

Supplement: Supplementary Figure 3 — Mean body weight of tumor bearing rats with implanted hepatic artery port-catheters receiving repeated infusions of saline or LDL nanoparticles over a 9 day study period. Arrows indicate time of HAI. [file Image_3.jpeg]

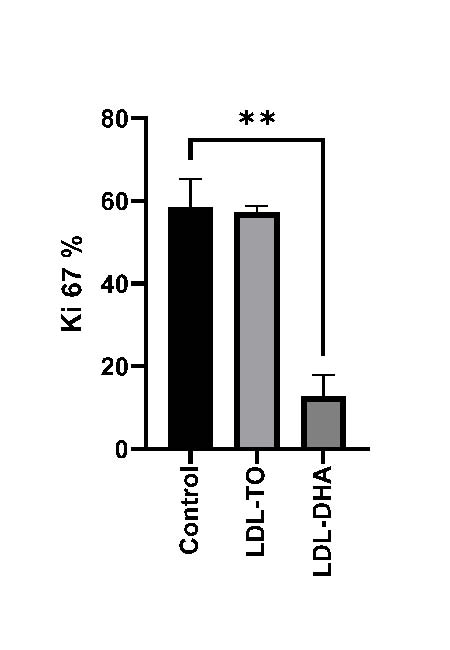

Supplement: Supplementary Figure 4 — Quantification of KI-67 immunohistochemistry in untreated controls, LDL-TO and LDL-DHA treated animals. Results are expressed as mean ± SEM. [file Image_4.jpeg]

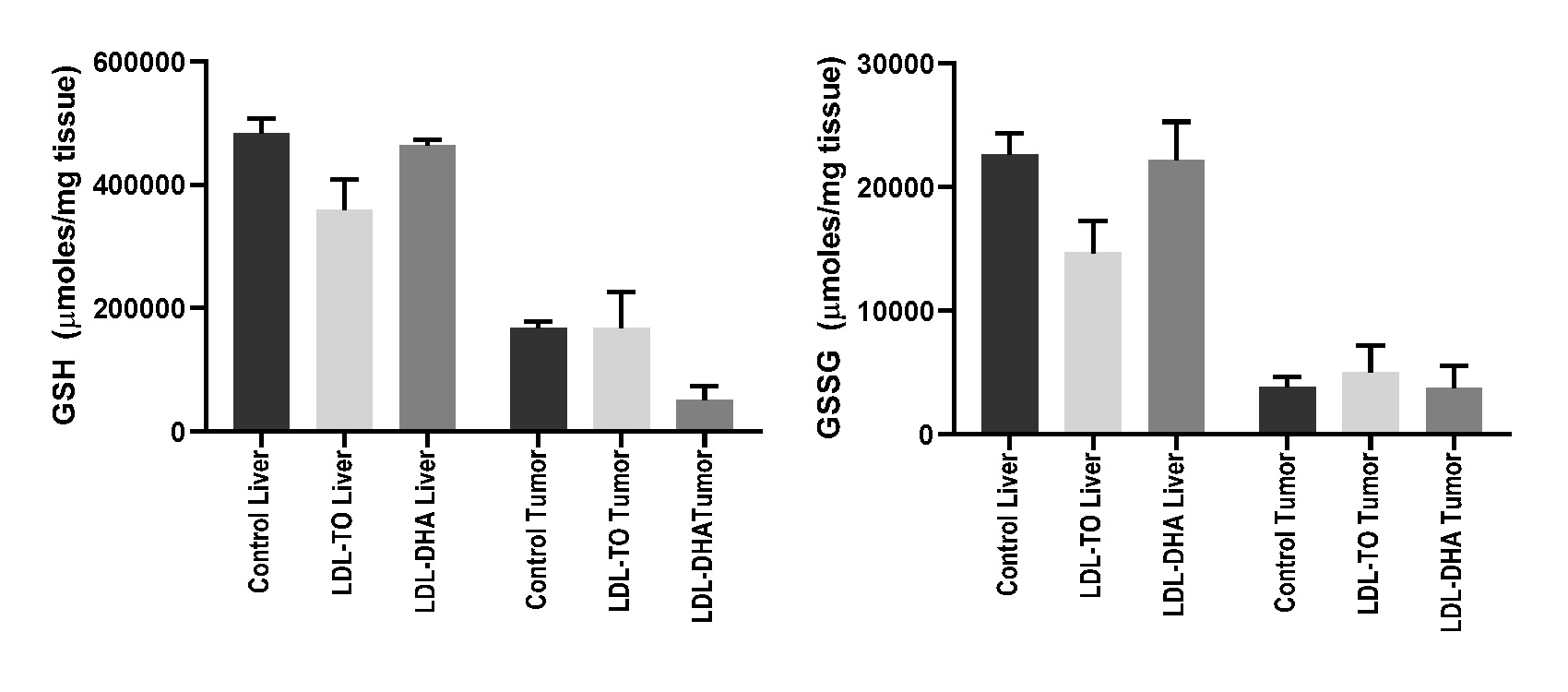

Supplement: Supplementary Figure 5 — Concentrations of reduced and oxidized glutathione in liver and tumor samples from untreated controls and rats following repeated HAI of LDL nanoparticles. The data is expressed as µmoles of glutathione per mg of tissue (mean ± SEM) for each treatment group. [file Image_5.jpeg]

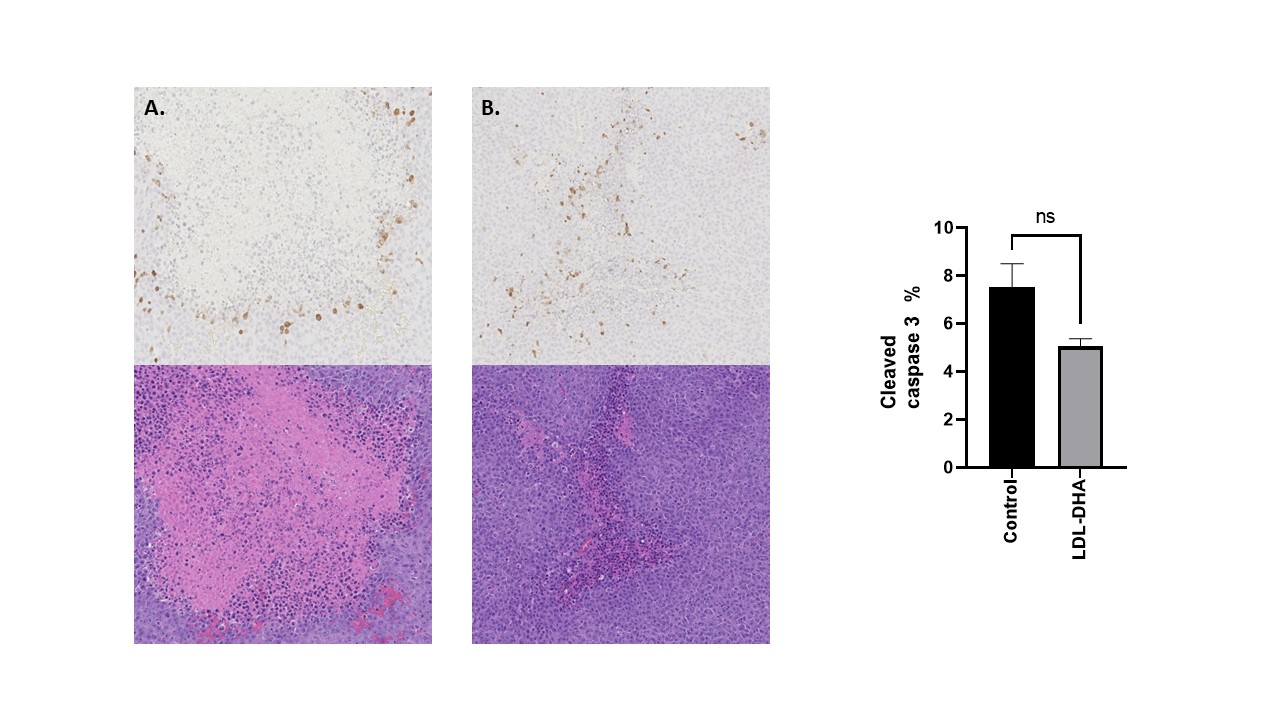

Supplement: Supplementary Figure 6 — H&E and Immunohistochemistry of N1S1 tumors after Sham and HAI treatment of LDL-DHA (2 mg/kg). N1S1 tumor tissue samples were collected 72 hours post-treatment. Corresponding tissue sections were stained for H&E and the apoptosis marker cleaved caspase-3. (A) Sham; (B) LDL-DHA. Images were taken at 20x magnification. Quantification of cleaved caspase-3 immunohistochemistry in untreated controls and LDL-DHA treated animals. Results are expressed as mean ± SEM. [file Image_6.jpeg]
