# Supplementary material for: Repeated trans-arterial treatments of LDL-DHA nanoparticles induce multiple pathways of tumor cell death in hepatocellular carcinoma bearing rats
Source: Front Oncol. 2022 Nov 24;12:1052221. doi: 10.3389/fonc.2022.1052221 (PMC9730405; doi:10.3389/fonc.2022.1052221)
Supplement: Supplementary file 7 [file DataSheet_1.pdf]

# Multiple Trans-arterial Treatments of Low-density Lipoprotein Docosahexaenoic Acid Nanoparticles in Hepatocellular carcinoma bearing Rats

Yuzhu Wang[1,2], Junjie Li[2], Goncalo Dias do Vale[3], Jaideep Chaudhary[2] , Arnida Anwar[2] , Jeffrey G. McDonald[3], Tao Qin[1] , Hongwei Zhang[1] and Ian R.Corbin[2,4,5] *

## SUPPLEMENTARY MATERIAL

### MATERIALS AND METHODS

***Preparation of LDL Nanoparticles.*** Apheresis plasma of patients with familial hypercholesterolemia was collected, LDL was isolated using sequential density gradient ultracentrifugation (22). Triolein (TO) and unesterified DHA (Nu-chek Prep, Inc) were incorporated into LDL by the reconstitution method as described previously (18).

***Cell Viability Test*** The N1S1 rat hepatoma cell line (ATCC, CRL-1603, Manassas, VA, USA) was cultured in Dulbecco's Modified Eagle's Medium (Sigma, D6429) supplemented with 10% fetal bovine serum and 1% penicillin-streptomycin. Cells were incubated at 37°C in a humidified environment containing 5% $CO_2$. For viability test, cells were seeded in 96-well plates with 30000 cells/well and grown to 80-90% confluency. Prior to treatment all cells were cultured in serum free media overnight (~18 hours). After respective treatments with LDL nanoparticles, cell viability was measured at 72 hours with water soluble tetrazolium salt, WST-8 (CCK-8, Dojindo Molecular Technologies). Briefly, cells were incubated with WST solution for 2 hours at 37°C. A ThermoMax M5 microplate reader was used to measure the absorbance at 450 nm. The relative cell viability was expressed as a percentage of the control.

*Chemical and cell death inhibitor studies*

To assess the pathway of LDL-DHA mediated cell death cell viability assays were also performed in the presence of selected cell death inhibitors (iron chelator, defiprone (DFP); caspase inhibitor, z-VAD-fmk; ferrroptosis inhibitor, liproxstatin-1 . All drugs were purchased from Selleck Chemicals. For this assay all cells were pretreated for 3 hours with the inhibitors prior to the addition of LDL-DHA. Chemicals or cell death inhibitors were used at the following concentrations: DFP, 20-40µM; Z-VAD-FMK, 50-100µM; liproxstatin-1, 50-200nM.

*Western Blot*

Samples were lysed in 1x cell lysis buffer (9803, Cell Signaling) and protein concentration was determined using Pierce™ BCA Protein Assay Kit (Thermo Fisher). Equal amounts of protein were loaded for each sample on a polyacrylamide gel and separated using electrophoresis. Thereafter, proteins were transferred to PVDF Transfer Membrane (Immobilon) and incubated overnight with primary antibodies against glutathione peroxidase 4 (GPX4) (1:500 dilution, sc-50497), Cleaved Caspase-3(1:1000 dilutions, Abcam ab2302) and β-actin (1: 1000 dilution, Cell Signaling sc-47778). Horseradish peroxidase-conjugated (HRP-conjugated) secondary antibodies were used and western blot signals were detected with ECL (Bio-Rad Laboratories).

*Methods of Lipid Peroxidation Measurement for N1S1 cells*

N1S1 cells (350,000) in 2 ml media without serum were plated on 6 well plate and incubated overnight. The next day, cells were stained with 1 uM Bodipy C11 581/591 dye (Invitrogen) for 30 minutes at 37° C. After staining, the cells were treated with different concentration of LDL-DHA for 24 hours. Non adherent cells in media were collected and attached cells were trypsinized. All cells were pooled together and then washed with PBS. Bodipy fluorescence of the cells were measured with flow

cytometry where green fluorescence indicated oxidized lipid species and red fluorescence indicated unoxidized lipid species. Lipid peroxidation is expressed as a ratio between green and red fluorescence sample signal multiplied by 100.

***Animal Studies***

All procedures involving animals were approved by the Institutional Animal Care and Use Committee of University of Texas Southwestern Medical Center. Eighteen Male, Sprague-Dawley rats, 7 weeks of age, were included in this study. All rats were housed in a temperature-controlled animal room (22± 2°C) under a 12h dark/light cycle, with access to laboratory food and water ad libitum (n=3 rats/plastic cage).

***Tumor Cell Inoculation.*** At the time of tumor cell inoculation rats were anesthetized with 2% isoflurane and a midline laparotomy incision (2cm) was made to expose the liver. N1S1 rat Hepatoma cells (1 x $10^7$) in a matrigel suspension was injected into the lower left lobe of the liver. Eight days post implantation tumor growth was monitored using Magnetic resonance image (MRI). Studies were initiated once the tumors reached a diameter of 1.0 - 1.5 cm (approximately 10-12 days post tumor inoculation).

***Surgical Placement of Indwelling Hepatic Arterial Infusion Port.*** Placement of indwelling hepatic arterial infusion port were performed in tumor bearing rats. Anesthetized rats were placed in supine position, and a midline incision (~5 cm) was made to enter the peritoneal cavity. Under a surgical microscope, the hepatic artery was exposed and ligated distal to gastroduodenal artery (GDA) with a 6-0 silk tie. A small arteriotomy was made on GDA and the polyurethane intravascular 2Fr tubing tip end (inner diameter 0.3mm, outer diameter 0.6mm; Access Technologies, Skokie, Illinois, USA) of the implantable infusion port (silastic PMIN port; Access Technologies, Skokie, Illinois, USA) was inserted at

the incision point on the GDA and advanced to the proper hepatic artery. The catheter was then secured by ligatures around the artery. Next a 1-2 cm pocket was created between the skin and muscle layer near the lower part of the abdominal incision to accommodate the implantable infusion port and the connected intra-arterial catheter. The implanted port was sutured and secured to the muscular fascia, followed by closure of the abdominal cavity using standard suture techniques. Thereafter, Taurolidine-Citrate Catheter lock Solution (TCS) (Access Technologies, Skokie, Illinois, USA) was injected into the port to ensure patency and lock the catheter. For the repeated hepatic artery infusion studies, LDL nanoparticles were injected percutaneously through the port, using a 24 gauge Huber point needle (Access Technologies, Skokie, Illinois, USA), followed by a TCS flush.

***Repeated Hepatic Arterial Infusions.*** Rats were randomly allocated into three groups: group I, untreated control rats (n=9); group II, LDL-TO controls (n=4) and group III, LDL-DHA (n=10). Repeated treatments of LDL nanoparticles was performed through the hepatic arterial infusion port at baseline and days 3 and 6. The repeated treatments of LDL-DHA was administered at a dose of 2 mg/kg (DHA) each. LDL-TO treatments were given at an equivalent dose to LDL-DHA. The dose and treatment frequency was selected as a 2mg/kg dose was previous demonstrated to be an effective therapeutic dose for treating tumors and a 3 day dosing frequency was selected as therapeutic responses to HAI of LDL-DHA nanoparticles are near complete in this time frame. Over the course of study the animals' body weights were monitored. All rats were sacrificed on the day 9 at which point blood and various organs were collected for histopathology and biochemical analyses.

***Magnetic resonance imaging (MRI)*** MRI was performed on all rats at baseline, 3, 6, and 9 days post treatment. A 9.4T MR imaging system (Varian/Agilent, Santa Clara, USA) was used to acquire images

with the following parameters: T2: TR/TEesp, 2500/10ms; echo-train length, 8; and T1: TR/TE 250/1.98ms, flip angle 70; the rest parameters of FOV, 64 × 64mm2; matrix size, 256 × 256; 18 slices without gap; section thickness= 2mm, averages= 6. For contrast-enhanced T1 weighted MRI, Magnevist (Bayer Schering Pharma AG, Berlin, Germany) was injected via the tail vein at a dose of 100 µL.

**Measurements of Tumor Volume, necrosis ratio and Tumor volume Doubling Time (DT)**

N1S1 Tumors appeared hypointense on T1 weighted images, and hyperintense on T2 and contrast-enhanced T1-weighted MR images. ImageJ software (National Institutes of Health, Bethesda, MD, USA) was used to measure the tumor size and necrosis ratio. Tumor area was measured on T1-weighted MR image, by manually circle the tumor lesion on all involved images with T2 image as reference. Tumor volume was calculated using the equation: tumor volume = Σ (tumor area on each slice × slice thickness). The degree of treatment induced tumor necrosis was measured from the contrast enhanced T1-weighted images. Successful transarterial treatment typically display radiographic features of a non-enhancing core region surrounded by a thin rim enhancement indicative of central tumor necrosis accompanied with a periphery of enhancing cells. The area of central non-enhancing tissue was delineated on ImageJ to estimate necrosis. The necrosis ratio was defined as the volume of tumor necrosis over that of the entire tumor volume, i.e. necrosis ratio = Σ (area of necrosis × slice thickness) / (area of whole tumor × slice thickness) × 100%. Necrosis ratio obtained from MRI was further validated with the corresponding histopathology findings.

***Histopathological analysis.*** At the time of euthanasia, partial excised liver, tumor, and spleen samples were collected, and were fixed with total immersion in 10% neutral buffered formalin for 24h, then embedded in paraffin, and sectioned into 5-µm slices and stained with Hematoxylin and Eosin (H&E). The slides were captured with an optical microscope (CX31, Olympus, Japan) at 100× magnification for

histopathology, and micrograph were taken with a microscope digital camera system (DP50, Olympus, Japan).

Necrosis Ratio determination

H&E slides of control and treated tumors were scanned using a histology slide scanner (PrimeHisto XE) and the images imported into ImageJ. The approximate area of the tumor was marked and measured. The image was then split into Red, Green and Blue Channels. The viable part of the tissue was most distinctive in the red channel. An intensity threshold was applied until only the viable area was selected. These areas were then selected with the Wand tool and measured. The necrosis tissue ratio was computed for each of the slides percentage necrotic index expressed as (necrotic area)/(total tissue area) × 100..

***Serum Collection and Analysis.*** Immediately following euthanization, 5mL of blood was collected from inferior vena cava in all rats, and was centrifuged at 2500 rpm for 10 min at 4˚C. Separated serum was collected into eppendorf tubes and stored at -20 ˚C until analysis. Plasma liver enzymes alanine aminotransferase (ALT), aspartate aminotransferase (AST), gamma glutamyl transferase (GGT), total bilirubin (TBIL), albumin (ALB), glucose (GLU), alkaline phosphatase (ALKP), Triacylglyceride (TRIG), direct high density lipoprotein (dHDL), Cholesterol (CHOL), creatinine (CREA) and blood urea nitrogen (BUN) were measured with a AU400e automated biochemical analyzer.

***Western Blot Analysis.*** Frozen samples liver or tumor tissue (~40mg) were homogenized in 500µL Cell Lysis Buffer (Cell signaling technology 9803) with EDTA-free Protease Inhibitor Cocktail (Roche) while on the ice. Samples were then centrifuged for 10 minutes at 14,000 rpm at 4°C, and the collected

supernatants were stored at −80 °C. Protein concentrations were measured by Pierce BCA Protein Assay Kit (prod#23227). Protein concentrations of the collected supernatants were determined using the Pierce BCA Protein Assay Kit (prod#23227). Then, equal amounts of sample (30 µg/lane) were separated on 10% SDS-PAGE and transferred onto Immobilon-FL PVDF membrane (Millipore, USA). After blocking with 5% nonfat milk, Western blotting was performed with primary antibodies against nuclear factor-κβ (NFκB-p65) (SC-514451), interleukin-6 (IL-6) (SC-57315), C reactive protein (CRP) (SC-69770) and glutathione peroxidase 4 (GPX4) (sc-50497) (all of 1:1,000, Santa Cruz) at 4 °C overnight, and then incubated with secondary antibodies (all of 1:2500 ) for 1 h at room temperature. β-Actin (sc-47778)(1:1,000, Santa Cruz) was used as a loading control. The protein bands were detected by ECL detection system and densitometry was quantified using ImageJ software (National Institutes of Health, MD).

***Lipid Peroxidation Analysis.*** Liver and tumor peroxidative damage was assessed by measuring the malondialdehyde (MDA) level in tissue sample using the thiobarbituric acid reactive substances (TBARs) assay as described previously.(23) Results were expressed as µM MDA formed/mg protein of tissue.

***GSH/GSSG Assay.*** Total soluble glutathione (GSH) and glutathione disulfide (GSSG) were measured in tissue homogenates using the enzymatic recycling method (24). Briefly, about 50mg of tissue were homogenized in 10 volumes of cold 5% metaphosphoric acid and 0.6% sulfosalicylic acid mixture. Protein was precipitated and the supernatant was used to determine GSH and GSSG. Results were expressed in µmoles per gram of tissue.

**Gas Chromatography-Mass Spectrometry Fatty Acid Analysis.** The gas chromatography-mass spectrometry (GC-MS) lipidomics analysis of fatty acids in the liver was adapted from the protocol by Val et al.(25).

**Chemicals and Materials**

All solvents were either HPLC or LC/MS grade and purchased from Sigma-Aldrich (St Louis, MO, USA).). Fatty acid (FA) standards (FA(16:0{$^2H_{31}$}), FA(20:4ω6{$^2H_8$}) and FA(22:6ω3{$^2H_5$}) purchased from Cayman Chemical (Ann Arbor, MI, USA), were used as internal standards. A mixture of twenty six fatty acids (GLC-490) were purchased from Nu-Chek Prep (Elysian, MN, USA), and used as reference standard for the GC-MS technique. All lipid extractions were performed in 16×100mm glass tubes with PTFE-lined caps (Fisher Scientific, Pittsburg, PA, USA). An eVol® precision pipette equipped with a glass syringe (Trajan Scientific, Austin TX , USA) was used for the addition of FA standards into the samples.

**Sample Preparation**

**Sample homogenization:** Approximately 100mg of rat liver or tumor was weighed and transferred to a 2.0-mL pre-filled Bead Ruptor tube (2.8mm ceramic beads, Omni International, Kennesaw, GA, USA), and 1mL of methanol/dichloromethane (1:2, v/v) was added. Tissue was homogenized with a Bead Ruptor (Omni International) for 50s (5.5 mps, 3 cycles, 10 sec/cycle, 5 s dwell time). The homogenates were transferred to glass tubes and diluted to a final concentration of 20 mg/mL using methanol/dichloromethane (1:2, v/v). Aliquots equivalent to 0.5mg of homogenized tissue, were transferred to fresh glass tubes for total and free fatty acid analysis.

**Total fatty acid analysis sample preparation:** The lipids were extracted by a three phase lipid extraction (3PLE). Briefly, 1mL of hexanes, 1mL of methyl acetate, 0.75mL of acetonitrile, and 1mL of water was added to the glass tube containing the sample. The mixture was vortexed for 5 seconds and then centrifuged at 2671×*g* for 5 min, resulting in separation of three distinct liquid phases. The upper (UP) and middle (MID) organic phases layers were collected into separate glass tubes and dried under $N_2$. The

dried extracts were resuspended in 1mL of 0.5M potassium hydroxide solution prepared in methanol, spiked with 100uL of 0.5µg/mL of fatty acid standards (FA(16:0{$^2H_{31}$}), FA(20:4ω6{$^2H_8$}) and FA(22:6ω3{$^2H_5$}), and hydrolyzed at 80°C during 60 minutes. Hydrolyzed fatty acids were extracted by adding 1mL each of dichloromethane and water to the sample in hydrolysis solution. The mixture was vortexed and centrifuged at 2671×*g* for 5 minutes, and the organic phase was collected to a fresh glass tube and dried under $N_2$.

**Fatty Acid profiling by GC-MS:** Total fatty acid profiles were generated by a modified GC-MS method *(Quehenberger et al., 2011)*. Briefly, dried extracts were resuspended in 50µL of 1% triethylamine in acetone, and derivatized with 50µL of 1% pentafluorobenzyl bromide (PFBBr) in acetone at room temperature for 25 min in capped glass tubes. Solvents were dried under $N_2$, and samples were resuspended in 500µL of isooctane. Samples were analyzed using an Agilent 7890/5975C (Santa Clara, CA, USA) by electron capture negative ionization (ECNI) equipped with a DB-5MS column (40m x 0.180mm with 0.18µm film thickness) from Agilent. Hydrogen (carrier gas) flow rate was 1.6mL/min and injection port temperature were set at 300°C. Sample injection volume was 1µL. Initial oven temperature was set at 150°C, and then increased to 200°C at a 25°C/min, followed by an increase of 8°C/min until a temperature of 300°C was reached and held for 2.2 minutes, for a total run time was 16.7 minutes. Fatty acids were analyzed in selected ion monotoring (SIM) mode. The FA data was normalized to the internal standards. Fatty acid with carbon length; $C \leq 18$ were normalized to FA(16:0{$^2H_{31}$}), $C = 20$ were normalized to FA(20:4 ω6{$^2H_8$}), and $C = 22$ were normalized to FA(22:6 ω3{$^2H_5$}). Data was processed using MassHunter software (Agilent).

**Immunohistochemistry**

To examine the contribution of early caspase mediate processes to LDL-DHA tumor killing N1S1-tumor bearing rats receiving a single transarterial infusion of LDL-DHA was examined 3 days post treatment.

Tumor tissue was excised and immunohistochemistry was performed as described previously using microwave antigen retrieval and cleaved caspase-3 (1:50 dilution; Abcam, Cambridge, MA. Tumor tissues from untreated tumor bearing rats serves as controls.

**Statistical Analysis**

Data were expressed as the mean ± standard error. Analysis of variance (ANOVA) with Tukey's multiple comparison post hoc testing was used for evaluation of differences between groups. Differences with a P value less than 0.05 was deemed statistical significance. All statistical analyses were performed using GraphPad Prism Version 5.0 (GraphPad Software, San Diego, CA).

**References**

Quehenberger, O., Armando, A.M., Dennis, E.A., 2011. High sensitivity quantitative lipidomics analysis of fatty acids in biological samples by gas chromatography-mass spectrometry. Biochim Biophys Acta **1811**, 648-656.
