# Supplementary material for: Repeated trans-arterial treatments of LDL-DHA nanoparticles induce multiple pathways of tumor cell death in hepatocellular carcinoma bearing rats
Source: Front Oncol. 2022 Nov 24;12:1052221. doi: 10.3389/fonc.2022.1052221 (PMC9730405; doi:10.3389/fonc.2022.1052221)
Supplement: Supplementary file 8 [file DataSheet_2.docx]

| **Serum parameters** | **Control** | **LDL-TO** | **LDL-DHA** |
| --- | --- | --- | --- |
| **ALT(µ/L)** | 40 ± 22 | 18 ± 6 | 36 ± 22 |
| **AST(µ/L)** | 273 ± 218 | 299 ± 241 | 175 ± 261 |
| **GGT(µ/L)** | 5.0 ± 0 | 5.0 ± 0 | 5.0 ± 0 |
| **TBIL(mg/dL)** | 0.2 ± 0.1 | 0.2 ± 0.1 | 0.2 ± 0.1 |
| **ALB(g/dL)** | 2.8 ± 0.3 | 2.5 ± 0.1 | 2.5 ± 0.2 |
| **ALKP(µ/L)** | 119 ± 27 | 128 ± 89 | 181 ± 50^Ψ^ |
| **GLU(mg/dL)** | 238±57 | 183±53 | 237 ± 41 |
| **TRIG(mg/dL)** | 112 ± 97 | 392 ± 165^ΨΨ^ | 51 ± 18 |
| **dHDL(mg/dL)** | 50 ± 12 | 30 ± 2 | 51 ± 11 |
| **CHOL(mg/dL)** | 100 ± 11 | 96 ± 12 | 90 ± 14 |
| **BUN(mg/dL)** | 14.3 ± 2.8 | 18.5 ± 2.1 | 18.6 ± 5.0 |
| **CREA(mg/dL)** | 0.4 ± 0.1 | 0.5 ± 0.2 | 0.4 ± 0.1 |

**Supplemental Table 1.** **Serum biochemistry of untreated control and rats repeated treated with LDL nanoparticles.** Data are expressed as mean ± SEM. Abbreviations: AST, aspartate aminotransferase; ALT, alanine aminotransferase; GGT, gamma glutamyltransferase; TBIL, total bilirubin; ALB, albumin; ALKP, alkaline phosphatase; BGLU, glucose; TRIG, triglycerides; dHDL, direct high density lipoprotein; CHOL, total cholesterol; UN, blood urea nitrogen; CREA, creatinine. ^Ψ^ Indicates difference with control group; ^Ψ Ψ^ indicates difference with control and LDL-DHA treated groups, P ≤ 0.05.

| Fatty Acid | Control | LDL-TO | LDL-DHA |
| --- | --- | --- | --- |
| FA (14:0) | 0.45 ± 0.08^a^ | 0.82 ± 0.09^a^ | 0.46 ± 0.05 ^a^ |
| FA (15:0) | 0.27 ± 0.04 ^a^ | 0.46 ± 0.07^a^ | 0.30 ± 0.03 ^a^ |
| FA (16:1 n7) | 0.57± 0.13 ^a^ | 1.40 ± 0.48^a^ | 0.66 ± 0.23 ^a^ |
| FA (16:0) | 62.83 ± 10.41 ^a^ | 88.66 ± 6.70^a^ | 67.91 ± 5.60 ^a^ |
| FA (18:3 n3) | 0.22 ± 0.04 ^a^ | 0.30 ± 0.11^a^ | 0.18 ± 0.02 ^a^ |
| FA (18:2n6) | 37.78 ± 6.33 ^a^ | 57.05 ± 5.91 | 34.34 ± 1.55 ^a^ |
| FA (18:1n9) | 12.25 ± 2.12 ^a^ | 19.77 ± 2.40 | 12.54 ± 1.48 ^a^ |
| FA (18:1n7) | 7.58 ± 0.82 ^a^ | 12.15 ± 3.34^a^ | 9.50 ± 1.18 ^a^ |
| FA (18:3n6) | 0.20 ± 0.05 ^a^ | 0.38 ± 0.08^a^ | 0.22 ± 0.07 ^a^ |
| FA (18:0) | 78.96 ± 11.18 ^a^ | 114.90 ± 8.61 | 79.44 ± 2.56^a^ |
| FA (20:4n6) | 53.92 ± 5.11 ^a^ | 60.95± 2.82 | 52.95 ± 1.70 ^a^ |
| FA (20:5n3) | 0.19 ± 0.02 ^a^ | 0.20 ± 0.06 | 0.20 ± 0.02^a^ |
| FA (20:3n3) | 1.00 ± 0.11 ^a^ | 1.04 ± 0.13^a^ | 0.94 ± 0.08 ^a^ |
| FA (20:2n6) | 0.86 ± 0.09 ^a^ | 0.73 ± 0.10^a^ | 0.88 ± 0.05 ^a^ |
| FA (20:1n9) | 0.47± 0.03 ^a^ | 0.45 ± 0.04 | 0.49 ± 0.03 ^a^ |
| FA (22:6n3) | 9.63 ± 1.18 ^a^ | 9.43 ± 2.03^a^ | 9.02 ± 0.81 ^a^ |
| FA (22:4n6) | 1.25 ± 0.123 ^a^ | 1.52 ± 0.24^a^ | 1.19 ± 0.10 ^a^ |
| FA (22:5n3) | 1.93 ± 0.19 ^a^ | 1.57 ± 0.16^a^ | 1.62 ± 0.21 ^a^ |
| FA (22:0) | 0.43 ± 0.07 ^a^ | 0.64± 0.05 | 0.49 ± 0.03 ^a^ |

**Supplementary Table 2**. Hepatic fatty acid composition of polar lipids from control, LDL-TO and LDL-DHA (2 mg/kg) treated rats. Data are expressed as mean ± SEM. ^a^ Means with different letters are significantly different from each other, P ≤ 0.05.

| Fatty Acid | Control | LDL-TO | LDL-DHA |
| --- | --- | --- | --- |
| FA (14:0) | 0.48 ± 0.11^a^ | 0.55 ± 0.00^a^ | 0.33 ± 0.12 ^a^ |
| FA (15:0) | 0.19 ± 0.04 ^a^ | 0.10 ± 0.04^a^ | 0.19 ± 0.07 ^a^ |
| FA (16:1 n7) | 0.79± 0.17 ^a^ | 0.51 ± 0.15^a^ | 0.47 ± 0.12 ^a^ |
| FA (16:0) | 27.54 ± 4.19 ^a^ | 25.26 ± 7.52^a^ | 17.78 ± 2.91 ^a^ |
| FA (18:3 n3) | 0.16 ± 0.03 ^a^ | 0.08 ± 0.01^a^ | 0.08 ± 0.01 ^a^ |
| FA (18:2n6) | 16.76 ± 2.17 ^a^ | 16.74 ± 8.11^a^ | 9.05 ± 1.43 ^a^ |
| FA (18:1n9) | 13.18 ± 1.87 ^a^ | 11.75 ± 6.41 | 8.29 ± 1.23 ^a^ |
| FA (18:1n7) | 1.43 ± 0.26 ^a^ | 2.37 ± 0.45^a^ | 1.19 ± 0.37 ^a^ |
| FA (18:3n6) | 0.37 ± 0.07 ^a^ | 0.20 ± 0.09^a^ | 0.19 ± 0.02 ^a^ |
| FA (18:0) | 15.40 ± 3.23 ^a^ | 9.74 ± 3.36 | 10.18 ± 2.62 ^a^ |
| FA (20:4n6) | 4.02 ± 0.40 ^a^ | 4.89± 0.21^a^ | 3.0 ± 0.53 ^a^ |
| FA (20:3n3) | 0.25 ± 0.02 ^a^ | 0.25 ± 0.05^a^ | 0.16 ± 0.02 ^a^ |
| FA (20:2n6) | 0.28 ± 0.04 ^a^ | 0.26 ± 0.05^a^ | 0.80 ± 0.09^b^ |
| FA (20:1n9) | 0.19 ± 0.02 ^a^ | 0.19 ± 0.04 | 0.14 ± 0.02 ^a^ |
| FA (22:6n3) | 0.64 ± 0.06 ^a^ | 0.65 ± 0.10^a^ | 0.50 ± 0.07^a^ |
| FA (22:4n6) | 0.71 ± 0.63 ^a^ | 0.65 ± 0.16^a^ | 0.58 ± 0.07 ^a^ |
| FA (22:5n3) | 0.36 ± 0.04 ^a^ | 0.25 ± 0.04^a^ | 0.24 ± 0.04^a^ |

**Supplementary Table 3**. Hepatic fatty acid composition of neutral lipids from control, LDL-TO and LDL-DHA (2 mg/kg) treated rats. Data are expressed as mean ± SEM. ^a^ Means with different letters are significantly different from each other, P ≤ 0.05.

| Fatty Acid | Control | LDL-TO | LDL-DHA |
| --- | --- | --- | --- |
| FA (14:0) | 0.66 ± 0.09^a^ | 0.83 ± 0.09^a^ | 0.54 ± 0.19^a^ |
| FA (15:0) | 0.26 ± 0.04 ^a^ | 0.47 ± 0.03^a^ | 0.29 ± 0.06 ^a^ |
| FA (16:1 n7) | 0.62± 0.12 ^a^ | 0.68 ± 0.19^a^ | 0.20 ± 0.16 ^a^ |
| FA (16:0) | 31.67 ± 3.29 ^a^ | 18.25 ± 2.78^b^ | 37.04 ± 5.18 ^a^ |
| FA (18:3 n3) | 0.02 ± 0.01 ^a^ | 0.06 ± 0.01^a^ | 0.02 ± 0.01 ^a^ |
| FA (18:2n6) | 9.43 ± 0.92 ^a^ | 12.76 ± 3.03^a^ | 7.14 ± 0.98 ^a^ |
| FA (18:1n9) | 12.89 ± 0.96 ^a^ | 11.89 ± 2.84 | 8.22 ± 1.02 ^a^ |
| FA (18:1n7) | 3.67 ± 0.58^a^ | 4.23 ± 0.68^a^ | 3.96 ± 0.25 ^a^ |
| FA (18:3n6) | 0.06 ± 0.01 ^a^ | 0.09 ± 0.02^a^ | 0.04 ± 0.00 ^a^ |
| FA (18:0) | 45.23 ± 5.98 ^a^ | 35.28 ± 1.74 | 44.91 ± 6.08 ^a^ |
| FA (20:4n6) | 13.98 ± 1.22 ^a^ | 12.91± 1.91^a^ | 13.03 ± 2.23 ^a^ |
| FA (20:5n3) | 0.07 ± 0.01 ^a^ | 0.07 0.01 | 0.05± 0.01^a^ |
| FA (20:3n3) | 0.34 ± 0.04 ^a^ | 0.34 ± 0.03^a^ | 0.29 ± 0.03 ^a^ |
| FA (20:2n6) | 0.49 ± 0.04 ^a^ | 0.81 ± 0.26^a^ | 0.51 ± 0.05 ^a^ |
| FA (20:1n9) | 0.47± 0.04 ^a^ | 0.38 ± 0.15 | 0.46 ± 0.09^a^ |
| FA (22:6n3) | 1.27 ± 0.17 ^a^ | 1.04± 0.15^a^ | 1.35 ± 0.29 ^a^ |
| FA (22:4n6) | 1.50 ± 0.22 ^a^ | 1.52 ± 0.30^a^ | 2.15 ± 0.51 ^a^ |
| FA (22:5n3) | 0.42 ± 0.05 ^a^ | 0.40± 0.06^a^ | 0.39 ± 0.05^a^ |
| FA (22:0) | 0.61 ± 0.13 ^a^ | 1.68 ± 0.59 | 0.65 ± 0.18 ^a^ |

**Supplementary Table 4**. Tumor fatty acid composition of polar lipids from control, LDL-TO and LDL-DHA (2 mg/kg) treated rats. Data are expressed as mean ± SEM. ^a^ Means with different letters are significantly different from each other, P ≤ 0.05.

| Fatty Acid | Control | LDL-TO | LDL-DHA |
| --- | --- | --- | --- |
| FA (14:0) | 0.26 ± 0.11^a^ | 0.53 ± 0.28^a^ | 0.70 ± 0.16^a^ |
| FA (15:0) | 0.10 ± 0.01 ^a^ | 0.14 ± 0.03^a^ | 0.26 ± 0.08 ^a^ |
| FA (16:1 n7) | 0.32± 0.05 ^a^ | 0.64 ± 0.24^a^ | 0.56 ± 0.11 ^a^ |
| FA (16:0) | 9.47 ± 4.55 ^a^ | 15.90 ± 4.59^a^ | 43.61 ± 9.57 ^b^ |
| FA (18:3 n3) | 0.03 ± 0.01 ^a^ | 0.04 ± 0.00^a^ | 0.04 ± 0.00 ^a^ |
| FA (18:2n6) | 3.84 ± 0.62 ^a^ | 5.64 ± 0.46^a^ | 8.35 ± 0.86 ^b^ |
| FA (18:1n9) | 5.89 ± 0.79 ^a^ | 6.24 ± 0.14 | 11.69 ± 2.64^a^ |
| FA (18:1n7) | 1.56 ± 0.42^a^ | 2.59 ± 0.45^a^ | 4.95 ± 1.05 ^b^ |
| FA (18:3n6) | 0.06 ± 0.01 ^a^ | 0.13 ± 0.07^a^ | 0.07 ± 0.01 ^a^ |
| FA (18:0) | 15.88 ± 5.44 ^a^ | 18.00 ± 2.59 | 48.39 ± 14.80 ^a^ |
| FA (20:4n6) | 3.55 ± 0.76 ^a^ | 5.15± 1.21^a^ | 12.34 ± 2.11 ^b^ |
| FA (20:3n3) | 0.19 ± 0.03 ^a^ | 0.28 ± 0.03^a^ | 0.47 ± 0.07 ^b^ |
| FA (20:2n6) | 0.37 ± 0.06 ^a^ | 0.65 ± 0.11^a^ | 0.80 ± 0.09 ^b^ |
| FA (20:1n9) | 0.40± 0.07 ^a^ | 0.58 ± 0.14 | 0.77 ± 0.26^a^ |
| FA (22:6n3) | 0.51 ± 0.16 ^a^ | 0.37± 0.05^a^ | 0.85 ± 0.08 ^b^ |
| FA (22:4n6) | 0.83 ± 0.15 ^a^ | 1.24 ± 0.32^a^ | 4.19 ± 0.71 ^b^ |
| FA (22:5n3) | 0.18 ± 0.03 ^a^ | 0.20 ± 0.02^a^ | 0.39 ± 0.05^b^ |

**Supplementary Table 5**. Tumor fatty acid composition of neutral lipids from control, LDL-TO and LDL-DHA (2 mg/kg) treated rats. Data are expressed as mean ± SEM. ^a^ Means with different letters are significantly different from each other, P ≤ 0.05.
